# Hydrogen Evolution Performance of a PdAu-Loaded Titanium Glycolate-Based Catalyst in Formic Acid Dehydrogenation with a High Turnover Frequency

**DOI:** 10.1021/acsomega.6c02175

**Published:** 2026-05-26

**Authors:** Razan Anwar Hamdan, Duygu Hacıefendioğlu, Burcu Gökçal Kapucu, Mustafa Polat, Ali Tuncel

**Affiliations:** † 37515 Hacettepe University, Chemical Engineering Department, Ankara 06800, Turkey; ‡ Hacettepe University, Department of Physics Engineering, Ankara 06800, Turkey

## Abstract

A superior titanium glycolate-based catalyst for thermo- and photocatalytic formic acid dehydrogenation (FADH) is synthesized for hydrogen evolution. Carbon-decorated mesoporous anatase TiO_2_ nanospheres (CTNSs) are obtained by the hydrolysis of titanium glycolate nanospheres (TGNSs). The selection of TGNSs as the precursor of the support allows varying the extent of carbon decoration, crystallinity, mean crystallite size, Ti­(III)/Ti­(IV) ratio, and oxygen vacancy of TiO_2_-based catalyst by controlling the hydrolysis time. A series of catalysts are obtained by immobilization of ultrafine PdAu nanoalloy onto amine-functionalized CTNSs with different crystalline properties and carbon contents. The nanocatalyst obtained using CTNSs with the lowest crystallinity (78.4%), smallest crystallite size (3.98 nm), and densely carbon-decorated anatase phase with a high oxygen vacancy (40.65%) and high Ti­(III)/Ti­(IIV) ratio (0.409) with a band gap energy of 1.255 eV (PdAu@ACTNSs-1) provides an exceptional TOF of 20,924 h^–1^ with 100% H_2_ selectivity in catalytic FADH at 60 °C. A TOF of 34,877 h^–1^ was obtained at 80 °C. TOFs up to 1507 h^–1^ are observed in visible-light-driven photocatalytic FADH at 25 °C via ^1^O_2_, ^•^OH radicals, and electron holes. PdAu@ACTNS-1 exhibits high stability over thermo-/photocatalytic FADHs. The synthetic route presents an innovative strategy for developing new high-performance catalysts for organic reactions.

## Graphical abstract

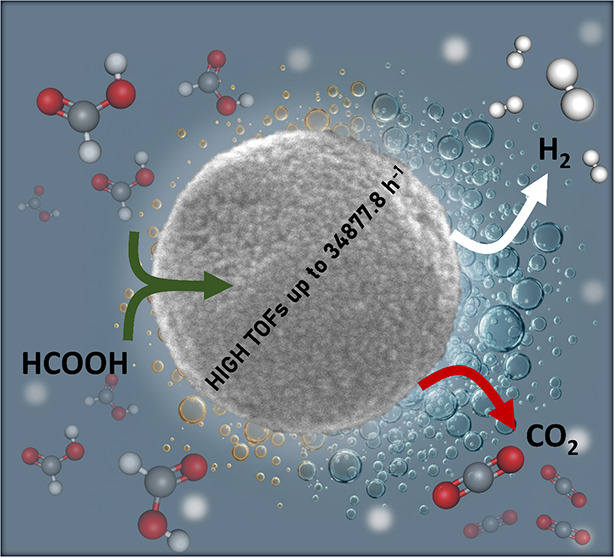

## 1 Introduction

In the pursuit of renewable and sustainable energy, hydrogen energy is a promising green energy source. The green nature of hydrogen energy arises from its clean combustion properties, producing only water as a byproduct without any greenhouse gas emissions, and hence decreases pollution and risks arising from climate change. Moreover, the long-term storage capacity of hydrogen makes it an ideal candidate to address seasonal and regional availability challenges. Its feasibility for integration into renewable energy infrastructures and ability to meet multiple industrial requirements make H_2_ a promising advancement in the global energy sector.^1–4^ The high flammability, continuous evaporation, and the use of expensive and complex equipment to store hydrogen in the physical state limit its practical usage. Thus, choosing an appropriate and suitable storage method for H_2_ with a potential for higher energy density is essential.^5–9^ Accordingly, chemical substances such as sodium borohydride,^10^ methanol,^11^ formic acid^12^ (FA), benzene, and cyclohexane,^6^ have been used as hydrogen carriers (HCs) and considered a better-suited choice for more convenient handling, storage, and transportation.^13,14^ At present, the most critical bottleneck for the practical application of these chemical hydrides is the development of highly active and economic catalysts to achieve efficient on-site hydrogen production. In this context, while non-noble transition metals, particularly Cu-based catalysts, have been comprehensively reviewed as promising and cost-effective alternatives for the dehydrogenation of various chemical hydrides,^15^ among all these options, FA is considered to be one of the best candidates for storing hydrogen due to its safety, biodegradability, low toxicity, practical storage, and transportation at room temperature.^5,16^ Also, its high H_2_ content (4.4 wt %) and convenient hydrogenation–dehydrogenation ability make it an ideal HC.^7^ FA can be decomposed according to the following reactions

1 $$\mathrm{HCOOH}\to H_{2}+{\mathrm{CO}}_{2}$$

2 $$\mathrm{HCOOH}\to \mathrm{CO}+H_{2}O$$

The dehydrogenation route in eq 1 is the desired decomposition path due to its hydrogen selectivity.^2,7^ The dehydration reaction exhibits decarbonylation, producing carbon monoxide, which is a harmful and toxic gas. Therefore, to attain an effective formic acid dehydrogenation (FADH) and ensure the reaction follows the dehydrogenation pathway, suitable catalysts must be selected to promote adsorption, facilitate charge transfer, and enable the necessary bond cleavages for hydrogen release.^17–19^

Various research groups have investigated different heterogeneous catalysts for the FADH reaction, and the results have shown that noble-metal nanoparticles, such as Pd, Ru, Ir, and Au, exhibit good catalytic performance for FADH under mild conditions. Among these options, Pd is considered to have the highest activity and selectivity as a monometallic catalyst.^3,17,19,21^ This experimental performance is also supported by theoretical modeling. For instance, spin-polarized density functional theory (DFT) calculations have demonstrated that Pd-catalyzed FADH exhibits a significantly lower effective energy barrier (0.76 eV) for the rate-determining step compared with other analogous metals such as Ni (1.03 eV) and Pt (1.56 eV).^21^ However, because Pd is regarded as an expensive metal, designing Pd-based catalysts with the incorporation of other metals is an alternative pathway for attaining efficient FADH.^20^ Tang et al. have developed Ni/Pd nanoparticles as efficient catalysts for FADH at room temperature. The involvement of the Ni core in the catalyst increases the activity of Pd, decreases the cost of the catalyst, and decreases the likelihood of CO formation.^22^ Zou et al. developed CdS-based nanocrystals surface-modified with nickel for the photocatalytic FADH. The presence of Ni modification enhances the adsorption of FA on the surface of the catalyst and also promotes the generation of hydroxyl radicals, which increases the hydrogen production rate.^23^ Although non-noble transition metals can be used for FADH, they yield relatively low TOF values, and their catalytic behavior is not very promising. Therefore, noble metals are a better choice for higher catalytic activity and H_2_ selectivity because of their superior properties.^2,20^

Au with a surface energy of 1.28 J m^–2^, which is lower than that of Pd (1.92 J m^–2^), is capable of increasing the stability of the catalyst, regulating its surface properties, and providing better dispersion of Pd nanoparticles (Pd NPs). The effect of surface charge polarization on Pd NPs promotes the adsorption of formate ions, and in the presence of Au, their rearrangement to a monodentate configuration is facilitated, thereby avoiding the formation of toxic CO.^17^ Accordingly, different kinds of supports have been investigated widely in this field to reinforce the electronic structure of the active catalysts and enhance the dispersion of metal nanoparticles to avoid their aggregation and deactivation during the reactions. Supports such as carbon-based materials, inorganic metal oxides, and metal–organic frameworks (MOFs) have been extensively investigated and utilized in this research area.^8,20^ Incorporating semiconductors in the supporting materials has been proven to show efficient and durable behavior to carry the active catalytic sites in order to provide electron transfer in the reaction, which is responsible for desorbing hydrogen atoms to form H_2_ molecules.^24^ Moreover, engineering amorphous–crystalline phases has emerged as a highly effective strategy in heterogeneous catalysis to modulate the local electronic structure, generate abundant defect sites (such as oxygen vacancies), and ultimately enhance the catalytic activity and synergistic effects.^25–28^

Heterogeneous catalysts for the thermal FADH reaction were synthesized by the immobilization of bimetallic AuPd nanoalloys onto different supports. In these runs, TOF values ranging between 1380 and 14,400 h^–1^ were achieved with different catalysts.^29–37^ The TOF values of 1896 and 3207 h^–1^ were obtained using AuPd nanoparticles (AuPd NPs) anchored on nitrogen-decorated carbon nanosheets, and nitrogen–carbon-doped TiO_2_ decorated with AuPd NPs as the catalysts in the thermal catalytic dehydrogenation of FA at 60 °C.^33,34^ Furthermore, recent studies have extensively demonstrated the versatility of advanced TiO_2_-based heterogeneous catalysts by significantly boosting the catalytic performance for FA.^38–40^ The nanocarbons decorated with PdAu NPs and N-doped TiO_2–*x*_ decorated with AuPd alloy provided maximum TOF values of 8355 and 8053 h^–1^ in thermal FADH at 60 °C.^35,36^ Modified cellulose nanocrystals immobilized with AuPd nanoalloy provided a TOF value of 4258 h^–1^ in FADH at 50 °C.^37^ Al-Qurahi et al. developed a Pd/Au bimetallic nanoalloy catalyst for efficient dehydrogenation of FA. Amine-functionalized forms of monodisperse–mesoporous SiO_2_, TiO_2_, and CeO_2_ microspheres, ca. 5.00 μm in size, obtained by staged-shape templated hydrolysis/condensation or decomposition protocols, were used as supports to immobilize bimetallic Pd/Au alloy.^30^ TOF values up to 3170 and 1391 h^–1^ were achieved in the thermal and photocatalytic dehydrogenations of FA, respectively. Chen et al. used carbon quantum dot-modified TiO_2_ as a support for immobilizing Pd nanoparticles to create an efficient catalyst for hydrogen production from FA.^31^ The catalyst was used in photocatalytic FADH and obtained a TOF value of 2666.1 h^–1^ at 35 °C with perfect hydrogen selectivity.^31^ A TOF value of 1380.5 h^–1^ was obtained with amine-functionalized mesoporous TiO_2_ decorated with Pd NPs due to the mesoporous nature of the catalyst, providing a higher mass transfer rate and efficient light absorption in the photocatalytic FADH reaction.^32^ Hence, TiO_2_ as a semiconductor is capable of providing electrons for the metal active sites attached to it, which leads to higher adsorption capacity of the formate dentates and a significant reduction in the energy barrier of H* desorption, which will, in total, give a synergistic effect together with the active sites for an efficient FADH. Moreover, the presence of amine functional groups can significantly increase the catalytic activity by anchoring the active sites, regulating the interactions between both the supports and metal nanoparticles, enhancing the O–H bond cleavage, and facilitating the desorption of adsorbed hydrogen atoms to form H_2_ molecules.^2^ More recently, the remarkable promoting effect of amine groups under light irradiation was further demonstrated by anchoring ultrafine PdAu nanoclusters onto amino-modified reduced graphene oxide. Benefiting from the strain and ligand effects in the alloy, alongside the Mott–Schottky effect between the metal and support, this catalytic system exhibited an exceptional TOF of 10699.5 h^–1^ at 298 K under visible light.^41^

In this work, a facile protocol was proposed for the synthesis of an exceptional catalyst for hydrogen evolution. Titanium glycolate nanospheres (TGNSs) of ca. 500 nm in size were synthesized using a modified protocol with titanium isopropoxide as the precursor. TGNSs were converted into densely carbon-decorated mesoporous anatase TiO_2_ nanospheres (CTNSs) via hydrolysis under reflux.^42,43^ CTNSs with different crystalline properties were obtained by changing the hydrolysis period of TGNSs and by sintering the obtained CTNSs. The heterogeneous catalysts obtained by the immobilization of bimetallic ultrafine PdAu nanoalloys onto the amine-functionalized forms of CTNSs with different crystalline properties were evaluated in both thermocatalytic and photocatalytic FADH reactions effectively. Here, we present the physical and chemical characteristics of the synthesized catalysts and their exceptional catalytic performance in thermocatalytic and photocatalytic FADH reactions.

## 2 Experimental Section

### 2.1 Materials

All commercial chemicals and reagents were of analytical grade and used as received without further purification. A complete list of materials and suppliers is provided in Section S2.1 of the Supporting Information.

### 2.2 Synthesis of Uniform, Mesoporous Titanium Glycolate Nanospheres (TGNSs)

The TGNSs were synthesized using a modified form of a published two-stage protocol.^42,43^ First, titanium isopropoxide (Ti-iPr, 0.5 mL) was added to ethylene glycol (EG, 40 mL) in a sealed bottle placed on a magnetic stirrer at 350 rpm. The mixture was stirred overnight at room temperature. Next, the Ti-iPr/EG solution was added to another solution obtained by adding DI water (1 mL) to acetone (200 mL). The resulting solution was magnetically stirred for 60 min to form TGNSs at room temperature. The product, TGNSs, was extensively washed with ethanol and DI water. The TGNSs were isolated by centrifugation at 5000 rpm for 3 min and vacuum-dried at 60 °C.

### 2.3 Synthesis of Mesoporous, Carbon-Decorated TiO_2_ Nanospheres (CTNSs)

Typically, TGNSs were dispersed in DI water (40 mL) by vortexing and ultrasonication. The dispersion was placed in the flask, which was connected to a condenser, and the system was placed in a thermostated oil bath with magnetic stirring. Heating was started by setting the temperature to 100 °C, and the dispersion was magnetically stirred for 1 h for the hydrolysis of TGNSs at the boiling point of water. The dispersion was cooled down to ambient temperature and centrifuged. Carbon-decorated TiO_2_ nanospheres (CTNSs-1) were precipitated, washed with DI water twice, and dried in a vacuum at 60 °C. The same protocol was followed with a longer reflux period (i.e., 2 h) for obtaining mesoporous, carbon-decorated TiO_2_ microspheres with higher crystallinity (i.e., CTNSs-2).

CTNSs-1, obtained with a reflux period of 1 h, were calcined in an air atmosphere at 450 °C for 4 h, with a heating rate of 2 °C/min to obtain CTNSs-3.

### 2.4 Functionalization by APTES and PdAu Loading onto TGNSs and ACTNSs

The functionalization of TGNSs and CTNSs by APTES, and the synthesis of the heterogeneous catalysts by loading PdAu ultrafine nanoalloys on ATGNSs and ACTNSs are explained in detail in Section S1.2 of the Supporting Information.

### 2.5 Thermocatalytic Formic Acid Dehydrogenation

PdAu bimetallic nanoalloy-loaded ATGNSs and ACTNSs-1, ACTNSs-2, and ACTNSs-3, with different crystalline properties, were used as catalysts for hydrogen generation using FA as the hydrogen precursor. For the preparation of the reaction medium, the catalyst (100 mg) was dispersed in DI water (4 mL) by ultrasonication for 1 min. The dispersion was transferred into an Erlenmeyer flask (100 mL), which was used as the reactor. The reactor was then sealed with a rubber septum attached to a thin hose to deliver the evolved gas into the measuring cylinder and placed into a temperature-controlled water bath, and placed on a magnetic stirrer. The reaction temperature was set between 30 and 80 °C. The other end of the hose was immersed in water placed in an inverted measuring cylinder to monitor the volume of gas evolved over time using the water displacement method. FA and SF solutions (0.5 mL, 5 M each) were injected into the reaction medium through a rubber septum when the required reaction temperature was reached in the reactor. At the moment when the two injections were completed, a stopwatch was started to track the volume change of the evolved gas with respect to time. The gas evolved from the reactor was analyzed using GC-MS (Agilent, 7890B, CA, U.S.A.).

Detailed descriptions of the catalytic testing setup and calculation of TOF values are provided in Section S1.8 of the Supporting Information.

### 2.6 Photocatalytic Formic Acid Dehydrogenation

A white COB LED (48.7 W, 4000 K) was used as the visible-light source to initiate the dehydrogenation at room temperature using PdAu bimetallic nanoalloy-loaded ACTNSs/ATGNSs as the catalyst. Typically, the catalyst (150 mg) was dispersed in 4 mL of DI water by ultrasonication and vortex mixing. The dispersion was transferred into the reactor, which was the same reactor used for the thermal dehydrogenation runs. The reactor was sealed and placed on a magnetic stirrer with one end of its hose inside and the other end immersed in water in a water-filled graduated cylinder. A distance of 15 cm was maintained between the reactor and the light source to achieve a 1 sun power density (100 mW cm^–2^). FA and SF solutions (0.5 mL, 5 M each) were injected through a rubber septum, and the light source was turned on. The volume readings were taken against time, as in the thermocatalytic test. The gas evolved from the reactor was analyzed using GC-MS. Detailed descriptions of the catalytic setup are provided in Section S1.9 of the Supporting Information.

## 3 Results and Discussion

### 3.1 Characterization of Titanium Glycolate-Based Nanospheres

A schematic representation of the synthetic protocol used for obtaining mesoporous titanium glycolate-based nanospheres with different crystalline properties is shown in Figure S1 of the Supporting Information. In the first stage, titanium glycolate nanospheres (TGNSs) were prepared using a modified protocol published previously, using titanium­(IV) isopropoxide and ethylene glycol as the precursors, with acetone and water as the solvents (Figure S1A of the Supporting Information).^42,43^ Two samples of mesoporous anatase TiO_2_ NSs, encoded as CTNSs-1 and CTNSs-2, were obtained by exposing TGNSs to thermal treatment with reflux times of 1 and 2 h, respectively (Figure S1B of the Supporting Information). On the other hand, CTNSs-1 obtained with a reflux period of 1 h were further oxidized via calcination in air at 450 °C to obtain CTNSs with higher crystallinity (i.e., CTNSs-3) (Figure S1B of the Supporting Information). Primary amine-functionalized mesoporous nanosphere samples (i.e., ATGNSs, ACTNSs-1, ACTNSs-2, ACTNSs-3) were obtained by covalent attachment of APTES to four distinct titanium glycolate-based nanospheres via a silanization reaction (Figure S1C of the Supporting Information). The heterogeneous catalysts (i.e., PdAu@ATGNSs, PdAu@ACTNSs-1, PdAu@ACTNSs-2, PdAu@ ACTNSs-3) were obtained by the immobilization of an ultrafine PdAu nanoalloy via NaBH_4_ reduction on amine-functionalized mesoporous nanospheres (Figure S1C of the Supporting Information).

The porosity characteristics of all the supports were determined by N_2_ adsorption–desorption analysis (Figure S2 of Supporting Information). All samples exhibited type IV isotherms with hysteresis loops, confirming their mesoporous nature (Figure S2A of the Supporting Information). The corresponding pore size distributions are shown in Figure S2B of the Supporting Information. Quantitative parameters such as the specific surface area, pore volume, and average pore diameter are documented in Table S1 of the Supporting Information. The TGNSs exhibited the highest surface area (333 m^2^ g^–1^) and the lowest mean pore size (3.4 nm). Specifically, increasing the heat treatment duration led to a gradual decrease in the specific surface area, accompanied by an enlargement of the pore size and pore volume. This evolution can be attributed to the progressive formation of oxide domains that drive pore coalescence. The pronounced drop in the surface area (60 m^2^ g^–1^) observed for the CTNSs obtained by calcination at 450 °C highlights the effect of high-temperature treatment on the collapse and coarsening of the porous framework.

The FTIR spectra of TGNSs, CTNSs-1, CTNSs-2, and CTNSs-3 are presented in Figure S3 of the Supporting Information. The band at 1073 cm^–1^, assigned to the C–O stretching vibrations, was exclusively observed in the TGNS spectrum, indicating the presence of glycolate species. The Ti–O–C stretching vibration appears at 1121 cm^–1^ for TGNSs and gradually shifts to 1125 and 1159 cm^–1^ for CTNSs-1 and CTNSs-2, respectively, before completely disappearing in CTNSs-3. This progressive disappearance suggests the cleavage of Ti–O–C linkages and the complete oxidation of organic residues with extended hydrolysis.^42,43^ The characteristic C=O stretching bands of acetone, observed at 1229 and 1269 cm^–1^ in TGNSs, vanished after hydrolysis, confirming the removal of residual solvent species.^42,43^ Similarly, the C–C stretching vibrations at 1357 and 1380 cm^–1^, with decreasing intensity with prolonged hydrolysis, were no longer detectable in CTNSs-3, indicating the breakdown of organic chains. The band at 1630 cm^–1^, present in TGNSs, CTNSs-1, and CTNSs-2, is primarily attributed to the bending vibration of adsorbed water molecules and surface hydroxyl (−OH) groups, which is further strongly supported by the broad O–H stretching band observed in the 3000–3600 cm^–1^ region. This band may also overlap with the signals of trace amounts of strongly chemisorbed organics.^42,43^ It diminishes and ultimately disappears upon calcination due to the thermal desorption of water and the decomposition of residual organics at elevated temperatures.^42,43^ The C–H stretching vibrations, initially located at 2864 and 2925 cm^–1^, exhibit red shifts to 2923 and 2968 cm^–1^, respectively, suggesting molecular rearrangements or partial cleavage of the C–H chains during the hydrolysis process.^42,43^

### 3.2 Characterization of Catalysts

SEM micrographs of the catalysts synthesized with different titanium glycolate-based nanospheres are shown in Figure 1A–D. As seen here, all catalysts were synthesized in the form of nanospheres with a narrow size distribution. The mean nanosphere size and coefficient of variation for the size distribution values of these catalysts, calculated using the SEM images with low magnifications in Figure 1A–D, are listed in Table 1. No significant changes were observed in either property by the heat treatment protocols. The TGNSs had a relatively smooth surface (Figure 1A). The SEM images showing the detailed surface morphology of the catalysts revealed that the surface roughness increased for the samples synthesized with reflux periods of 1 and 2 h (Figure 1B,1C). A catalyst with a considerably rough surface was obtained after a reflux period of 1 h and calcination at 450 °C (Figure 1D). The SEM image of plain CTNSs-1 is shown in Figure S4 of the Supporting Information.

**Figure 1:**
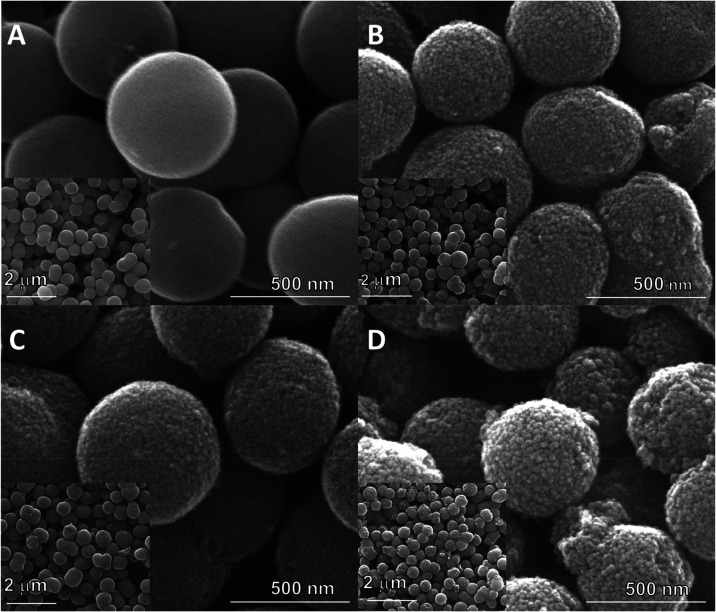
Electron microscopic characterization of catalysts: SEM images of (A) PdAu@TGNSs, (B) PdAu@ACTNSs-1 with a reflux period of 1 h, (C) PdAu@ACTNSs-2 with a reflux period of 2 h, and (D) PdAu@ACTNSs-3 with a reflux period of 1 h and calcination at 450 °C for 4 h. Magnification: 200.000*x;* for insets, 50.000*x*.

**Table 1:** Size Characteristics and Noble Metal Contents of the Heterogeneous Catalysts

catalyst	average particle size (nm)	CV (%)	Pd content (wt %)	Au content (wt %)
PdAu@ATGNSs	560	8.6	2.3 ± 0.1	2.1 ± 0.1
PdAu@ACTNSs-1	510	5.3	2.1 ± 0.1	2.5 ± 0.1
PdAu@ACTNSs-2	582	6.5	2.1 ± 0.1	2.1 ± 0.1
PdAu@ACTNSs-3	470	4.5	1.14 ± 0.01	1.62 ± 0.03

The HRTEM characterization of PdAu@ACTNSs-1 is shown in Figure 2A–H. PdAu@ACTNSs-1 was synthesized with a narrow size distribution, as also observed by SEM (Figure 2A). The SAED pattern in Figure 2B clearly demonstrates the typical crystal planes of the anatase phase in PdAu@ACTNSs-1. The *d*-spacing values in Figure 2B are consistent with the Miller indices for anatase. Figure 2C,D shows the coexistence of the PdAu nanoalloy with the amorphous and crystalline regions of PdAu@ACTNSs-1, marked by red and blue circles, respectively. To further investigate the structural composition, HAADF-STEM imaging coupled with EDAX spectroscopy was performed specifically on the amorphous regions clearly observed on the surface of PdAu@ACTNSs-1. The HAADF image and the corresponding EDAX spectrum are shown in Figure S5 of the Supporting Information. Elemental analysis revealed that these regions predominantly consisted of Ti, confirming the existence of an amorphous titania phase that formed an amorphous–crystalline phase boundary. The PdAu nanoalloy layer was clearly visualized using bright-field and dark-field TEM imaging, as shown in Figure 2E,F, respectively. Furthermore, the EDAX line-scan spectra presented in Figure 2G, which are consistent with the dark-field TEM observation, not only demonstrate that the metallic species penetrated through the mesoporous surface of ACTNSs-1 up to ca. 40 nm, but also reveal a highly correlated colocalization of the Pd and Au signals. Furthermore, point EDAX spectroscopy performed on individual nanoparticles confirmed the simultaneous presence of both Pd and Au within a single particle (Figure S6 of the Supporting Information). This local elemental verification provides direct evidence of successful nanoalloy formation. In the high-resolution TEM image (Figure 2H), highly dispersed ultrafine PdAu nanoparticles are distinctly observed as dark spheres anchored on the support. To evaluate the size distribution and ensure statistical reliability, a particle size histogram was constructed by measuring >100 individual nanoparticles randomly selected from three different TEM micrographs (Figure S7 of the Supporting Informatio**n**). The mean particle diameter was calculated to be 1.81 ± 0.48 nm, revealing the ultrasmall nature of the nanoalloy.

**Figure 2:**
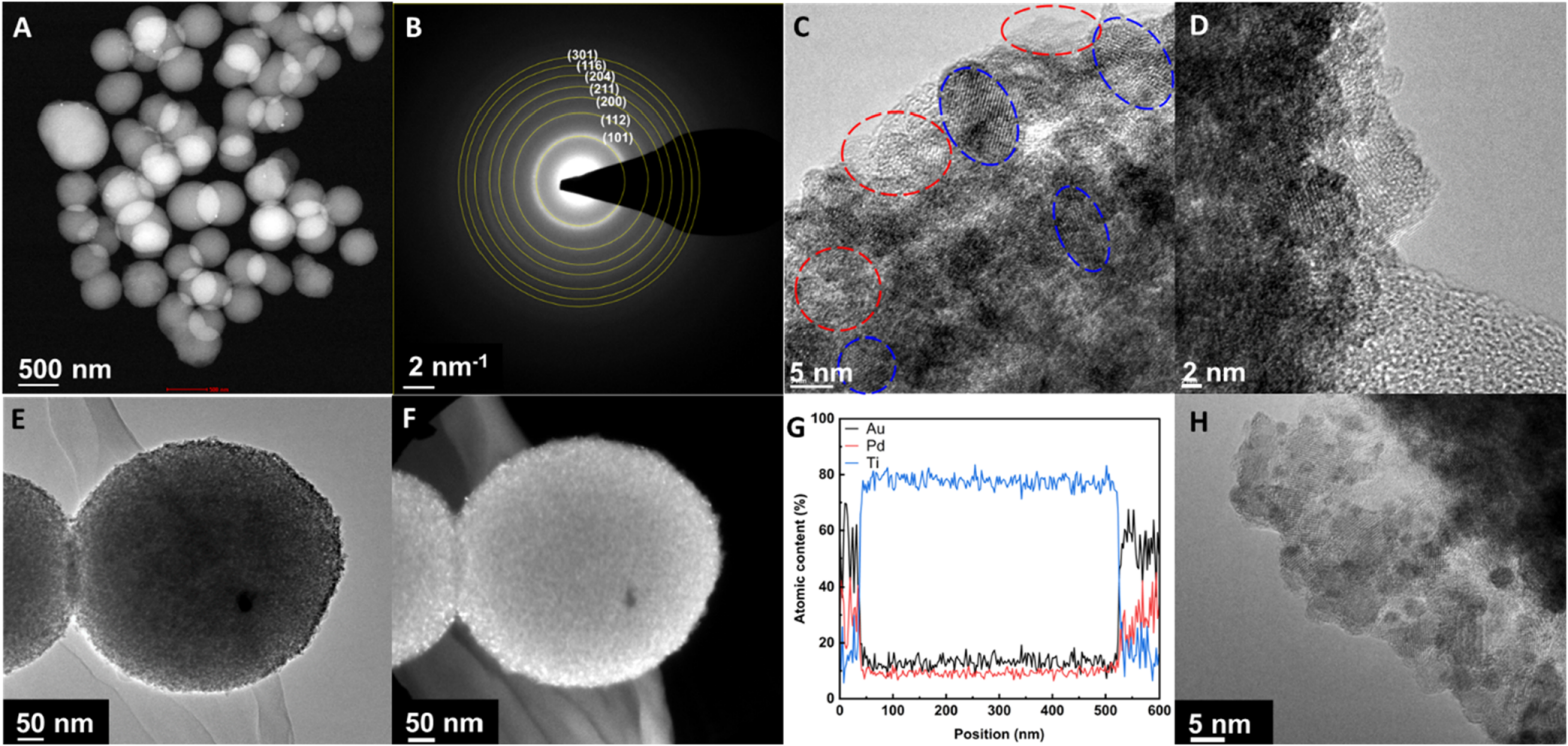
Characterization of PdAu@ACTNSs-1 by HRTEM: (A) TEM image with low magnification, (B) SAED pattern, (C), (D) HRTEM images showing amorphous and crystalline regions, (E) bright-field HAADF image showing PdAu layer, (F) dark-field HAADF image showing PdAu layer, (G) EDAX line scan showing PdAu shell, and (H) TEM image showing PdAu nanoalloy on NSs.

In the catalyst synthesis, the feed concentrations of Pd and Au were 2.5 wt %, with the total PdAu feed concentration fixed at 5.0 wt % with respect to the mesoporous nanospheres. The final precious metal contents of the heterogeneous catalysts determined by ICP-OES analysis are presented in Table 1. The determined Pd and Au contents of PdAu@ATGNSs, PdAu@ACTNSs-1, PdAu@ACTNSs-2, and PdAu@ACTNSs-3 were close to the feed concentrations of the corresponding metals. The lower Pd and Au contents of PdAu@ACTNSs-3 may be explained by their reduced surface hydroxyl concentration due to the calcination of the support at 450 °C, which in turn lowers the APTES and metal bindings onto the corresponding support.

DLS analysis was used to determine the hydrodynamic size and hydrodynamic size distribution of PdAu@ACTNSs-1 in both DI water and the FADH reaction medium. The average hydrodynamic particle diameter of PdAu@ACTNSs-1 was measured to be 503 nm in DI water (Figure S8A of the Supporting Information), while in the reaction medium, it was observed to be 502 nm (Figure S8B of the Supporting Information). These values align well with the mean size observed in the SEM image (i.e., 510 nm in Figure S8B of the Supporting Information) for PdAu@ACTNSs-1. This suggests that no significant agglomeration occurs in the FADH reaction medium with the selected catalyst.

The crystallographic properties of the catalysts were investigated using X-ray diffraction (XRD) spectroscopy (Figure 3). The TGNS-based catalyst displayed no distinct peaks, consistent with its amorphous character. In contrast, sharper and more intense peaks emerged for the catalysts produced with the supports subjected to prolonged thermal treatment, indicating improved crystallinity. All the crystalline samples were identified as anatase TiO_2_, with characteristic peaks at 2Θ = 25.3°, 38.0°, 47.9°, 54.5°, 62.8°, 69.0°, and 75.1°, corresponding to the (101), (112), (200), (211), (204), (116), and (301) planes, respectively (JCPDS 01-075-2544). The crystallite sizes calculated using the Scherrer equation are given in Table 2, along with the *d*-spacing and lattice constant values. The percent crystallinity of the obtained catalysts was calculated as the ratio of the integrated area under the crystalline diffraction peaks to the total area of the diffraction pattern. As expected, no specific peak belonging to PdAu NPs immobilized on mesoporous NSs was observed due to their low size.^44^

**Figure 3:**
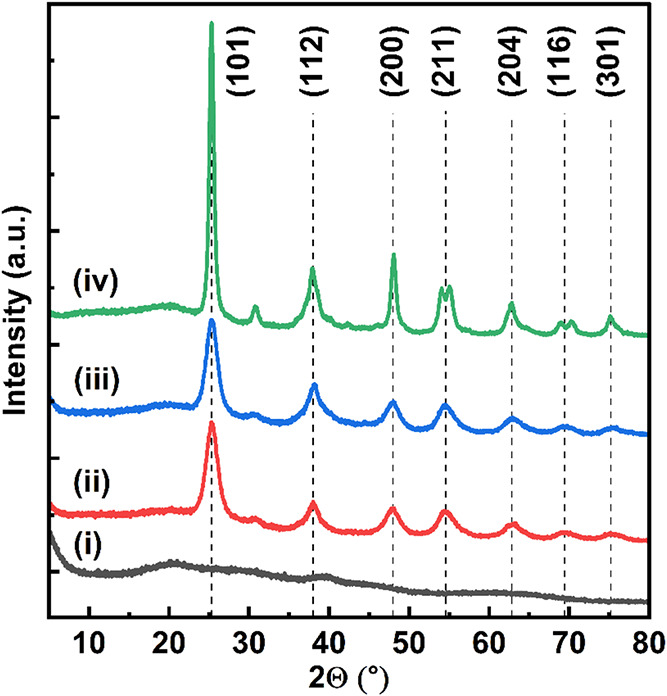
XRD patterns of (i) PdAu@TGNSs, (ii) PdAu@ACTNSs-1, (iii) PdAu@ACTNSs-2, and (iv) PdAu@ACTNSs-3.

**Table 2:** Crystalline Properties of the Heterogeneous Catalysts

XRD evaluation of crystalline properties						
				lattice constants (nm)		
catalyst	treatment	crystallite size (nm)	*d*-spacing (nm)	*a*	*c*	crystalinity (%)
PdAu@ACTNSs-1	1 h reflux	3.98	0.194	0.3823	0.9380	78.4
PdAu@ACTNSs-2	2 h reflux	4.16	0.194	0.3820	0.9374	81.4
PdAu@ACTNSs-3	1 h reflux + calcination	9.30	0.194	0.3831	0.9448	89.9

XRD analysis revealed that the duration of the thermal treatment had a direct impact on the crystallite size of the support. CTNSs-1, obtained after the shortest heat treatment period (1 h), exhibited the smallest crystallite size (Table 2). Extending the treatment to 2 h produced TiO_2_ with an average crystallite size of 4.16 nm, whereas additional calcination further promoted crystal growth, yielding an average crystallite size of 9.30 nm (Table 2). This progressive increase confirms that calcination facilitates the coalescence of the crystallites, resulting in larger and more ordered domains. Importantly, the *d*-spacing values remained constant across all samples, indicating that the anatase phase of TiO_2_ was preserved in the heat-treated supports (i.e., CTNSs-1, CTNSs-2, and CTNSs-3). Nevertheless, slight variations were observed in the lattice parameters, particularly along the *a*-axis, suggesting subtle distortions associated with the thermal treatment. For the *c*-axis, CTNSs-3 displayed the highest value, consistent with the lattice rearrangement induced by calcination. Such shifts may also reflect changes in the defect concentration within the crystal lattice. The degree of crystallinity exhibited a clear trend in the following order (Table 2)­

CTNSs−1<CTNSs−2<CTNSs−3

The pronounced increase for CTNSs-3 underscores that calcination not only drives crystal growth but also reduces the amorphous fraction, leading to a more ordered phase. Overall, these findings highlight that calcination results in larger crystallites, enhanced crystallinity, and slight lattice expansion, whereas CTNSs-1 and CTNSs-2 retain smaller crystallites and relatively lower crystallinity.

The surface chemistry of the catalyst, PdAu@ACTNSs-1, containing crystalline anatase with amorphous domains, was analyzed using XPS spectroscopy (Figures 4 and 5A,B). The survey spectrum confirmed the presence of the Au 4f, C 1s, Pd 3d, Ti 2p, and O 1s signals at binding energies of 83.05, 284.50, 335.03, 458.51, and 529.56 eV, respectively (Figure 4A). The high-resolution spectra of the Ti 2p, O 1s, and C 1s core levels are presented in Figure 4B–D. Deconvolution of the Ti 2p spectrum revealed four peaks at 457.97, 458.51, 463.36, and 464.42 eV, assigned to Ti­(III) 2p_3/2_, Ti­(IV) 2p_3/2_, Ti (III) 2p_1/2_, and Ti­(IV) 2p_1/2_, respectively. Quantitative analysis yielded a Ti­(III)/Ti­(IV) ratio of 0.409, indicative of abundant defect sites associated with oxygen vacancies on PdAu@ACTNSs-1 (Figure 4B). The O 1s spectrum was resolved into three components at 529.56, 530.15, and 531.98 eV, attributed to lattice oxygen, oxygen vacancies, and chemisorbed oxygen, respectively (Figure 4C). The oxygen vacancy/lattice oxygen ratio was calculated to be 40.7%, which closely matched the Ti­(III)/Ti­(IV) ratio. This strong correlation indicates that the Ti­(III) centers predominantly arise from oxygen vacancy formation in the PdAu@ACTNSs-1 lattice. Finally, the C 1s spectrum displayed peaks at 284.50, 286.04, and 287.99 eV, corresponding to C–C, C–O, and Ti–O–C species, respectively. The peak for Ti–O–C is an evidence of carbon decoration onto the TiO_2_ lattice (Figure 4D).

**Figure 4:**
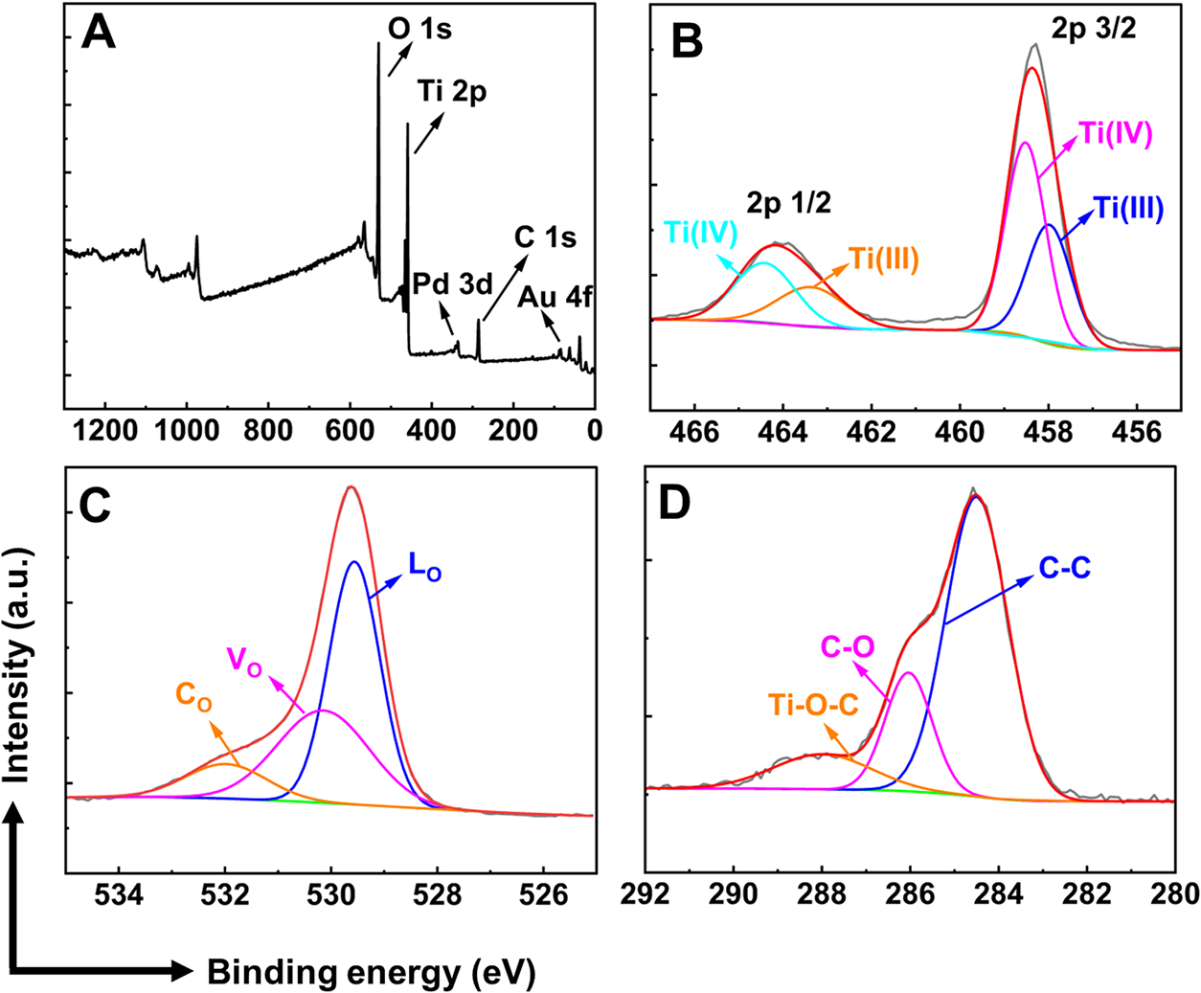
XPS spectra of PdAu@ACTNSs-1: (A) survey analysis, core-level spectra for (B) Ti 2p, (C) O 1s, and (D) C 1s scans.

**Figure 5:**
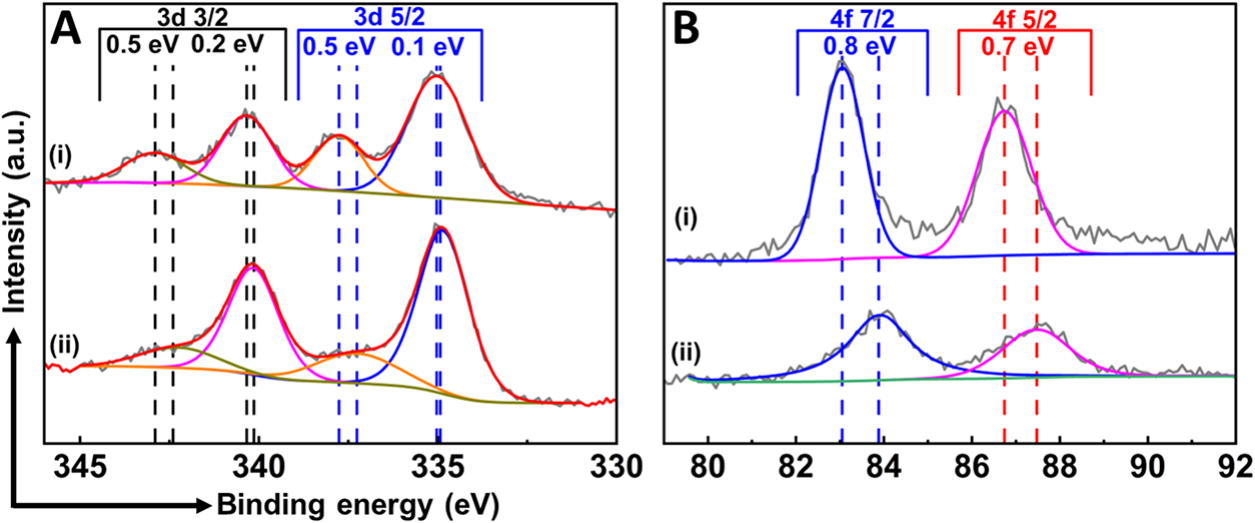
High-resolution core-level XPS spectra comparing the bimetallic and monometallic catalysts: (A) Pd 3d spectra of (i) PdAu@ACTNSs-1 and (ii) Pd@ACTNSs-1; (B) Au 4f spectra of (i) PdAu@ACTNSs-1 and (ii) Au@ACTNSs-1.

The Pd 3d spectrum showed four peaks at 335.03, 337.76, 340.34, and 342.90 eV, corresponding to the Pd 3d_5/2_ and Pd 3d_3/2_ states (Figure 5A­(i)). The Au 4f spectrum exhibited characteristic peaks at 83.05 and 86.74 eV, assigned to Au 4f_7/2_ and Au 4f_5/2_, respectively, confirming the presence of metallic Au (Figure 5B­(i)). To analyze the nanoalloy formation between the Pd and Au components of the active site, monometallic catalysts supported by ACTNSs (i.e., Pd@ACTNSs-1 and Au@ACTNSs-1) were prepared. The core-level spectra of the Pd 3d scan with Pd@ACTNSs-1 are shown in Figure 5A­(ii). Here, clear downshifts of 0.1 and 0.5 eV are observed in the binding energies of the two deconvoluted Pd 3d_5/2_ peaks of Pd@ACTNSs-1 compared to the corresponding ones in the Pd 3d scan of PdAu@ACTNSs-1. Similar downshifts of 0.2 and 0.5 are also obtained with the two deconvoluted peaks of Pd 3d_3/2_ of Pd@ACTNSs-1 when the Pd 3d scan of PdAu@ACTNSs-1 is considered. The core-level spectra obtained for the Au 4f scan with Au@ACTNSs-1 are shown in Figure 5B­(ii). Clear upshifts of 0.7 and 0.8 eV are observed in the binding energies of the Au 4f_7/2_ and Au 4f_5/2_ peaks of Au@ACTNSs-1, respectively, compared to the corresponding ones in the Au 4f scan of PdAu@ACTNSs-1. Consequently, all of these shifts demonstrated the formation of the PdAu nanoalloy on ACTNSs-1.^30,45,46^

The survey XPS spectra and core-level spectra for the Ti 2p, O 1s, and C 1s scans for PdAu@ATGNSs, PdAu@ACTNSs-2, and PdAu@ACTNSs-3 are also shown in Figure S9 of the Supporting Information. The surface characteristics of the catalysts using the core-level spectra for the Ti 2p, O 1s, and C 1s scans in Figures 4 and S9 of the Supporting Information are listed in Table 3. The XPS results revealed that the Ti­(III)/Ti­(IV) ratio and oxygen vacancy values of PdAu@ACTNSs-1 and PdAu@ACTNSs-2 were higher than those of amorphous PdAu@ATGNSs (Table 3). In other words, hydrolysis via heat treatment of the titanium glycolate-based support under reflux allowed the synthesis of catalysts with higher Ti­(III)/Ti­(IV) ratios and higher oxygen vacancies (i.e., PdAu@ACTNSs-1 and PdAu@ACTNSs-2). The increase in the reflux period of the TGNSs from 1 to 2 h allowed the synthesis of a catalyst with the highest Ti­(III)/Ti­(IV) ratio and highest oxygen vacancy (i.e., PdAu@ACTNSs-2). However, the lowest Ti­(III)/Ti­(IV) ratio and the lowest oxygen vacancy values were obtained with the catalyst supported by TGNSs subjected to refluxing and calcination at 450 °C. To further investigate the presence of oxygen vacancies and Ti^3+^ species, electron spin resonance (ESR) spectroscopy was performed on the PdAu@ATGNSs, PdAu@ACTNSs-1, PdAu@ACTNSs-2, and PdAu@ACTNSs-3 catalysts at 290 K. As shown in Figure S10 of the Supporting Information, strong and distinct paramagnetic resonance signals were detected at magnetic fields of 3377 and 3485 G. The corresponding g-factors were calculated as 2.067 and 2.003, respectively. The g-factor of 2.003 is attributed to oxygen vacancies within the catalysts.^47–50^ In addition to oxygen vacancies, a signal with a g value of 2.067 was detected. Because the ESR measurements were conducted in the dark under an ambient air atmosphere, this specific signal is attributed to chemisorbed oxygen radicals on the catalyst surface.^51–53^ The defected Ti^3+^ sites within the catalysts exhibit a strong chemical affinity for atmospheric oxygen. Ambient O_2_ molecules chemisorb onto these vacancies and interact with the surrounding surface oxygen framework. This electron transfer process generates highly reactive paramagnetic species (such as O^–^) due to the surface defect chemistry.^52^ Most importantly, the PdAu@ACTNSs-1 catalyst exhibited the highest intensity for the *g* = 2.067 signal. To determine the carbon content of the catalysts, CHNS elemental analysis was conducted, and the results are presented in Tables 3 and S2 of the Supporting Information. The results showed that PdAu@ATGNSs had the highest carbon content, as expected. The applied heat treatment protocols effectively decomposed the organic precursors. While PdAu@ATGNSs exhibited a carbon content of 7.45 wt %, the thermal treatment significantly reduced the carbon content to 3.50, 3.74, and 1.83 wt % for PdAu@ACTNSs-1, PdAu@ACTNSs-2, and PdAu@ACTNSs-3, respectively (Table 3). The lowest crystallite size (3.98 nm), the highest Ti­(III)/Ti­(IV) ratio (0.409), and the highest oxygen vacancy (40.65%) are the other remarkable properties of PdAu@ACTNSs-1, which synergistically enhance the strong metal–support interactions crucial for its superior catalytic performance.

**Table 3:** Surface Characteristics of the Heterogeneous Catalysts

catalyst	treatment	Ti(III)/Ti(IV) ratio^a^	oxygen vacancy (%)^a^	*C* content (wt %)^a^
PdAu@ATGNSs	no treatment	0.334	24.53	7.45
PdAu@ACTNSs-1	1 h reflux	0.409	40.65	3.50
PdAu@ACTNSs-2	2 h reflux	0.544	53.58	3.74
PdAu@ACTNSs-3	1 h reflux + calcination	0.212	23.31	1.83

a The Ti­(III)/Ti­(IV) ratio and oxygen vacancies were calculated based on the XPS spectra, and the *C* content (wt %) was obtained via CHNS analysis.

The Tauc plot was used to determine the band gap energy of the selected catalyst, PdAu@ACTNSs-1. For this analysis, a dilute dispersion of the catalyst in DI water was prepared, and the dispersion was analyzed using a UV–vis spectrophotometer between 190 and 1100 nm. A plot of absorbance against wavelength is estimated from the device, which later allows for the calculation of the band gap energy using eq 3, where a is the optical absorption coefficient, A is a constant, hv is the photon energy, and *E*_g_ is the band gap energy. The Tauc plot is estimated by plotting (a*h*v)^2^ against *h*v.

3 $${\left(\mathrm{ahv}\right)}^{2}=A\left(\mathrm{hv}-E_{g}\right)$$

Figure S11 of the Supporting Information shows the Tauc plot for PdAu@ACTNSs-1, which is the catalyst providing the best performance in the thermocatalytic FADH run. Hence, a band gap energy of 1.255 eV was calculated for PdAu@ACTNSs-1.

### 3.3 Catalytic Dehydrogenation of Formic Acid

#### 3.3.1 Thermocatalytic FADH Runs

Four distinct titanium-based nanospheres, derived from a titanium glycolate precursor and subsequently subjected to different postsynthesis treatments, were employed as supports to anchor and stabilize the active sites, thereby providing structural frameworks for catalytic functionality (i.e., PdAu@TGNSs, PdAu@ACTNSs-1, PdAu@ACTNSs-2, PdAu@ACTNSs-3). The effect of the titanium glycolate-based support type used in the synthesis of heterogeneous catalysts on the kinetics of the thermocatalytic FADH reaction is shown in Figure 6.

**Figure 6:**
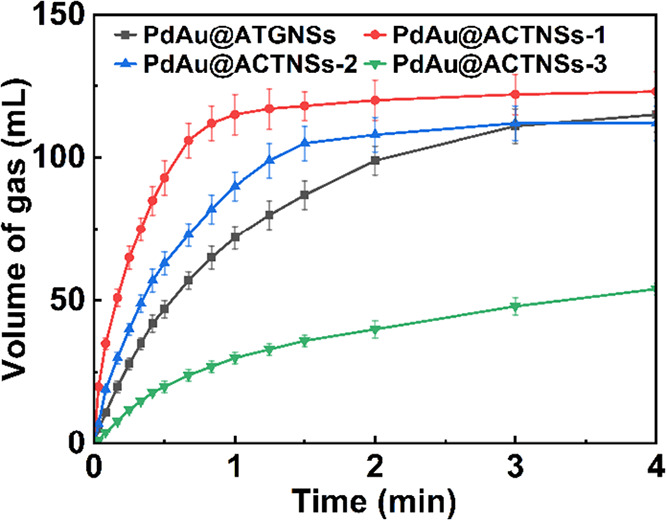
Effect of titanium glycolate-based support type used in the catalyst synthesis on the kinetics of the thermocatalytic FADH reaction. Conditions (unless otherwise stated): catalyst concentration: 20 mg mL^–1^, Pd/Au weight ratio: 1/1, PdAu loading 5.0 wt %/wt., FA/SF mole ratio: 1/1, 60 °C, stirring: 350 rpm in the dark.

The catalyst supported by mesoporous anatase-type TiO_2_ NSs prepared using a heat treatment period of 1 h (i.e., PdAu@ACTNSs-1) exhibited the best catalytic performance by the completion of FADH within 1 min (Figure 6). In order to check the selectivity, the GC-MS spectrum of the gas sample evolved in the thermocatalytic FADH reaction using PdAu@ACTNSs-1 as the catalyst was recorded (Figure S12 of the Supporting Information). As shown, a clear spectrum, including a strong CO_2_ signal at *m*/*z* of 44 and a very weak signal for water vapor at *m*/*z* of 18.1, was obtained. No significant signal for CO was observed in the gas evolved from the FADH reaction. These findings clearly demonstrate that the thermocatalytic FADH reaction with PdAu@ACTNSs-1 takes place with 100% H_2_ selectivity. The catalyst utilizing mesoporous anatase-type TiO_2_ as the support with an extended hydrolysis time of 2 h (i.e., PdAu@ACTNSs-2) also demonstrated notable catalytic performance, where the reaction ended after 3 min, indicating fast and efficient catalytic performance. The titanium glycolate-supported catalyst (i.e., PdAu@ATGNSs) ranked third and exhibited a relatively lower performance than the anatase-type and mesoporous titanium dioxide-supported catalysts. The reaction was completed in 3.5 min, which still represents competitive performance when current similar catalysts are considered.^29,30,33–36^ In contrast, the catalyst utilizing supports that experienced both calcination and water boiling treatments (PdAu@ACTNSs-3) exhibited relatively lower activity and failed to produce the expected volume of H_2_, indicating poor catalytic performance.

A comparison of the TOF values obtained with different titanium glycolate-based catalysts (calculated based on the total molar amounts of Pd and Au determined by ICP-OES) with those of the current catalysts developed for the thermocatalytic FADH reaction is presented in Table 4. In this work, the highest initial TOF (calculated at 20% FA conversion) for the thermocatalytic FADH reaction at 60 °C reached 20924 h^–1^ over the PdAu@ACTNSs-1 catalyst. This performance was achieved with mesoporous TiO_2_ NSs possessing the lowest crystallite size (3.98 nm) and the lowest crystallinity (78.4%) among the tested anatase-containing catalysts (i.e., PdAu@ACTNSs-2 and PdAu@ACTNSs-3) (Table 4). A smaller crystal size results in the formation of a higher number of defective regions, thereby enhancing the catalytic performance.^44^ The use of the same catalyst in the FADH reaction at 80 °C provided a TOF value of 34,877.8 h^–1^, as demonstrated by the parametric FADH runs. These TOF values are much higher than those of the current heterogeneous catalysts developed for the thermocatalytic FADH reaction (Table 4).

**Table 4:** Comparison of TOF Values Obtained with Different Titanium Glycolate-Based Catalysts with Those of the Current Heterogeneous Catalysts in the Thermocatalytic FADH

catalyst	temperature (°C)	TOF (h^–1^)^a^	refs
PdAu@ACTNSs-1	60	20924.0	this work
PdAu@ACTNSs-1	80	34877.8	this work
Ni_0.8_Mo_0.2_/ZIF-67@SiO_2_	25	13183	^60^
Au_4_Pd_6_/N-TiO_2–*x*_	60	8053	^36^
PdAu@ACTNSs-2	60	7774.8	this work
Pd_0.6_Au_0.4_/VXC-74-NH_2_	25	7385	^61^
Au_2_Pd_8_/TiO_2_–NC-800	60	6798	^34^
Ag_2_Pd_8_/TiO_2_	60	4789	^62^
PdAu@TGNSs	60	4729.5	this work
Au_0.4_Pd_0.6_/PEI–PDA@CNCs	50	4258	^41^
Pd/NHPC-AC	60	4115	^55^
Pd_0.8_Au_0.2_/UiO-66-(NH_2_)_2_	50	3660	^36^
Au_2_Pd_8_/TiO_2_–NC-800	60	3207	^34^
PdAu@SiO_2_	60	3170	^30^
PdAu@ACTNSs-3	60	2417.0	this work
AuPd/n-CNS-Th-160	60	1896	^33^
Au–Pd/SBA-15-Amine	50	1786	^63^
Pd_0.5_Au_0.5_@AC	40	1648	^64^
Pd/Co-CN	30	1403	^65^
Au_1_Pd_3_/BNNFs-A	25	1181.1	^66^
R- Au_6_Pd_4_@CB	25	1075	^67^
Ni_0.4_Pd_0.6_/NH_2_–N-rGO	25	954.3	^68^
Ag_1_Pd_4_@NPC	80	936	^69^
PdNMC-400	25	913	^70^
PdAu/C–P	50	808.6^a^	^71^
Pd@CeO_2_	40	807.7	^72^
PdAu-MnO_*x*_/N-SiO_2_	25	785	^73^
AuPd/TiO_2_(L)-400	25	592	^74^
Au_0.3_Pd_0.7_-(La_2_O_3_)_0.6_/CNTs	50	589	^75^
AuPd/C	50	230	^76^
AgPd@Pd/TiO_2_	60	143.77	^77^

a The initial TOF was calculated as 20% of the FA conversion.

The catalyst prepared using mesoporous TiO_2_ NSs with slightly higher crystallite size and higher crystallinity (PdAu@ACTNSs-2) provided a lower TOF (i.e., 7774.8 h^–1^) than PdAu@ACTNSs-1. The catalyst prepared using the mesoporous anatase-type TiO_2_ support with the highest crystallinity and highest crystallite size (PdAu@ACTNSs-3) provided the lowest TOF (Tables 1 and 4). When the catalysts prepared using crystalline anatase-type TiO_2_-based supports were considered, the TOF of the thermocatalytic FADH reaction decreased with increasing crystallinity and crystallite size of the support. The lowest Pd and Au contents and the lowest surface area of the support are the apparent reasons for the lowest TOF (2417 h^–1^) obtained with the catalyst produced with hydrothermally treated and calcined TiO_2_ NSs. On the other hand, the catalyst prepared using TGNSs with a completely amorphous character (PdAu@ATGNSs) provided a lower TOF (i.e., 4729.5 h^–1^) than those prepared with mesoporous TiO_2_ NSs with relatively low crystallite size and low crystallinity (i.e., PdAu@ACTNSs-1 and PdAu@ACTNSs-2) (Tables 1 and 4).

Both the TOF obtained with PdAu@ACTNSs-1 and the current catalysts, providing TOF values higher than 4000 h^–1^ (Table 4) indicated that carbon doping into the inorganic matrix of the catalyst is a critical factor for providing high catalytic activity in thermocatalytic FADH.^29,34,54–56^ In the core-level spectra for the C 1s scan of PdAu@ACTNSs-1, the peak at the binding energy of 288.0 eV is assigned to the Ti–O–C bond (Figure 4D). However, no peak that may be attributed to the Ti–C bond at a binding energy of 280.4 or 280.6 eV was observed in the same spectra. The existence of a Ti–O–C bond together with the absence of a Ti–C bond is a typical indicator of a carbon-decorated TiO_2_ matrix found in PdAu@ACTNSs-1.^57–59^ Indeed, the selection of a titanium glycolate-based starting material for obtaining anatase-containing mesoporous TiO_2_ NSs should be the reason for the carbon decoration within the TiO_2_ matrix by heat treatment.

By evaluating the physical and chemical characterization results of all catalysts synthesized, the exceptional catalytic performance indicated by the high TOF value can be attributed to several factors: (i) the lowest crystallinity (78.4%) and (ii) the smallest crystal size among the anatase-based catalysts (3.98 nm), (iii) a high Ti­(III)/Ti­(IV) ratio on the surface (0.409), (iv) high surface oxygen vacancy (40.7%), and (v) the ultrafine PdAu nanoalloy, which is finely dispersed on a large surface area (268 m^2^ g^–1^), with an appropriate surface concentration within the structure of the catalyst termed as PdAu@ACTNSs-1. Briefly, the catalyst with the aforementioned properties exhibited the highest TOF among the catalysts obtained from Ti-glycolate-based nanospheres. These findings highlight the critical role of the supporting material in imparting a synergistic improvement in the catalytic activity.

In order to determine the appropriate catalyst concentration for thermocatalytic FADH, the catalyst exhibiting the best performance (AuPd@ACTNSs-1) was selected, and its concentration varied between 10 and 40 mg mL^–1^ (Figure 7A). It was observed that higher catalyst concentrations yielded faster reactions. However, a catalyst concentration of 20 mg mL^–1^ provided the highest TOF in all the runs (Table 5). Accordingly, a catalyst concentration of 20 mg mL^–1^ was selected as the appropriate concentration for the other FADH runs.

**Figure 7:**
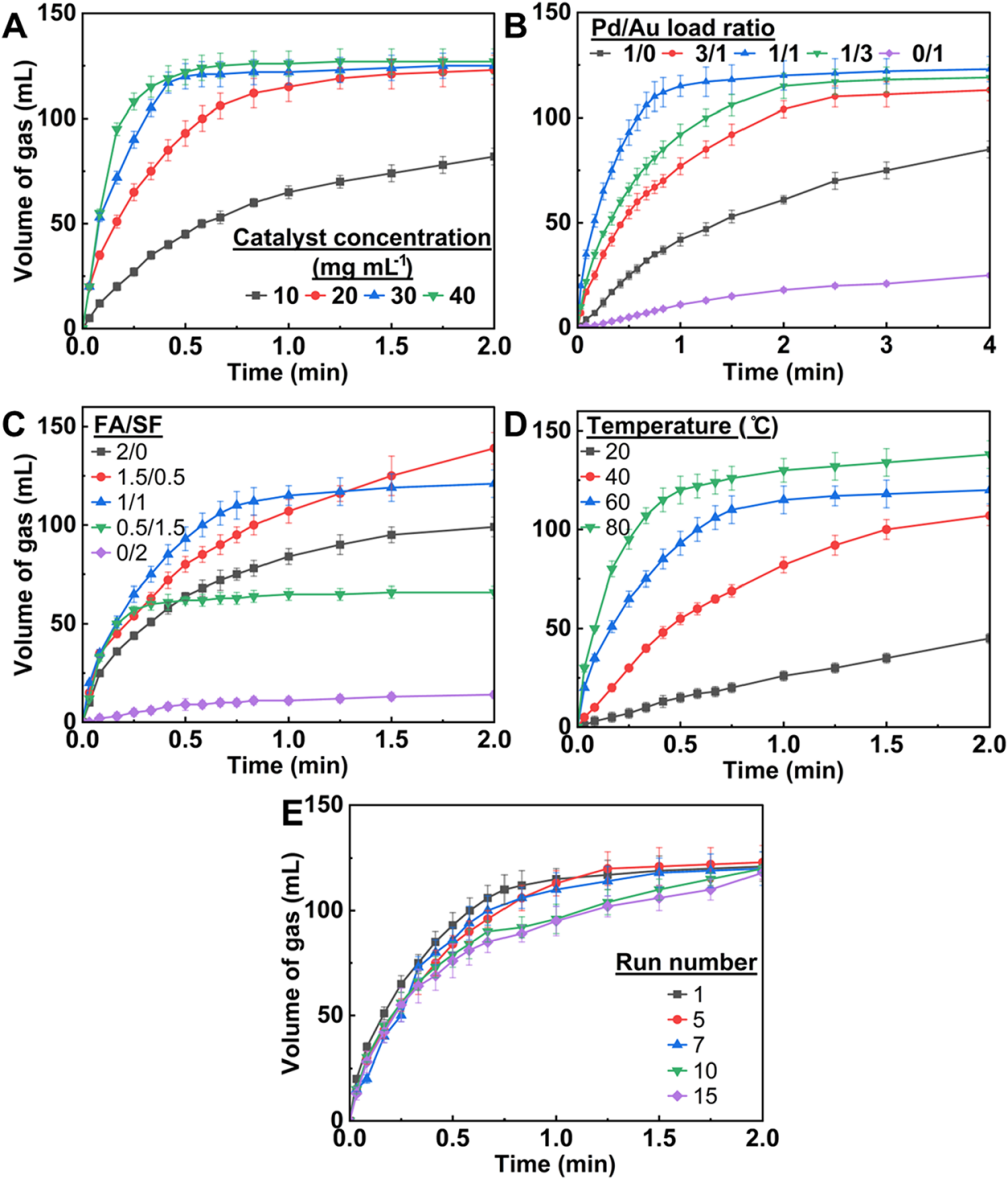
Effect of (A) catalyst concentration, (B) Pd/Au atomic ratio, (C) FA/SF ratio, and (D) temperature on the kinetics of the thermocatalytic FADH reaction using PdAu@ACTNSs-1 as the catalyst. (E) Reusability of PdAu@ACTNSs-1 in the thermocatalytic FADH reaction. Conditions (unless otherwise stated): catalyst concentration: 20 mg mL^–1^, Pd/Au weight ratio: 1/1, PdAu loading 5.0 wt %/wt., FA/SF mole ratio: 1/1, temperature: 60 °C, stirring: 350 rpm in the dark.

**Table 5:** TOF Values Obtained under Different Reaction Conditions in the Thermocatalytic FADH Using PdAu@ACTNSs-1 as the Catalyst^a^

PdAu@ACTNSs-1 concentration (mg mL^–1^)	TOF (h^–1^)
10	8796.2
20	20924.0
30	15632.3
40	11788.6
FA/SF ratio (mol/mol)	TOF (h^–1^)
2/0	11522.8
1.5/0.5	16651.3
1/1	20924.0
0.5/1.5	14839.9
0/2	138.6
temperature (°C)	TOF (h^–1^)
20	1011.0
40	4597.6
60	20924.0
80	34877.8

a Conditions (unless otherwise stated): catalyst concentration: 20 mg mL^–1^, Pd/Au weight ratio: 1/1, PdAu loading: 5.0 wt %/wt., FA/SF mole ratio: 1/1, 60 °C, stirring: 350 rpm in the dark.

Different Pd/Au atomic ratios with a total feed ratio of 5 wt % were examined to determine the catalytic activity. The reaction kinetics observed for each test are shown in Figure 7b. When 5 wt % of either Pd or Au was used individually as monometallic catalysts, the observed catalytic performance was markedly low. Specifically, the catalyst containing 5 wt % Pd required approximately 40 min to achieve complete conversion, while the 5 wt % Au catalyst failed to achieve full FA conversion within the same period. These results indicated that monometallic systems exhibited poor catalytic activity. When a bimetallic combination was used, the catalyst activity improved. For instance, when the weight ratio of Pd/Au was 3/1, a better catalytic performance was obtained, and the reaction ended after 4 min, whereas for a loading weight ratio of 1/3, the reaction ended in 2 min. The optimal catalytic performance was achieved with a Pd/Au weight ratio of 1/1, corresponding to an equal metal loading of 2.5 wt % for both Pd and Au. This balanced distribution appears to promote synergistic interactions between the two metals, thereby enhancing the overall catalytic activity. The higher catalytic activity observed when using a bimetallic combination is assigned to the synergistic interactions evolving between the two metals arising from the regulated surface energies and properties.^27,28,34^

In another set, FADH runs were conducted by changing the molar ratio of FA/SF between 2/0 and 0/2 (Figure 7C) to systematically elucidate the role of SF in the reaction system. In the absence of SF (FA/SF = 2/0), the catalytic performance decreased, and the expected final H_2_ volume was not obtained. Mechanistically, this is because FA alone is a weak acid with poor dissociation, resulting in a small number of reactive formate intermediates on the catalyst surface. When the molar ratio of FA/SF was 1.5/0.5, the catalyst exhibited better catalytic performance due to the added formate ions; however, the FADH reaction was slow to reach the final, expected H_2_ volume. The use of an FA/SF molar ratio of 1/1 resulted in superior catalytic activity, and the reaction was completed in less than 1 min. At this optimal ratio, the system acts as a highly effective buffer, providing an abundant supply of HCOO^–^ for rapid adsorption while maintaining sufficient proton (H^+^) availability for efficient H_2_ evolution.^29^ Decreasing the molar ratio of FA/SF to 0.5/1.5 also resulted in a fast reaction; however, it yielded a lower TOF value of 14839.9 h^–1^ (Table 5). This decline can be attributed to the competitive adsorption of excess formate ions blocking the active Pd sites or a relative deficiency in the proton supply required for the final H_2_ recombination step.^29^ Considering these results, an appropriate FA/SF mole ratio was chosen as 1/1.

As shown in Figure 7D, the rate of thermocatalytic FADH generation increases with increasing reaction temperature. For instance, at 40 °C, the reaction proceeded slowly and required approximately 15 min to complete, whereas at 20 °C, the reaction did not even yield the expected volume of hydrogen. As expected, the TOF values increased with increasing reaction temperature (Table 5). The highest TOF in this work was 34877.8 h^–1^ at 80 °C.

Examining the effect of temperature on the catalytic behavior also allowed the calculation of the activation energy of the catalyst, as derived from the Arrhenius plot (Figure S13A of the Supporting Information). By utilizing the slope value from this figure, the apparent activation energy was calculated to be *E*_a_ = 45.22 kJ mol^–1^. This value is close to the activation energies for catalyzing FA dehydrogenation found in the literature.^62,78^ An Eyring plot was also constructed to provide information about the apparent activation enthalpy and apparent activation entropy, which were calculated to be 42.54 kJ mol^–1^ and −108.8 J mol^–1^ K^–1^, respectively (Figure S13B of the Supporting Information).

In order to investigate the stability of PdAu@ACTNSs-1, 15 consecutive runs were performed for thermocatalytic FADH under optimized conditions (Figures 7E and S14 of the Supporting Information). As seen here, the catalyst maintained superior performance in thermocatalytic FADH, achieving 100% conversion of FA. ICP-MS analysis of the catalyst after five cycles provided Pd and Au contents of 2.2 ± 0.1% and 2.0 ± 0.1%, respectively. This finding demonstrates that no significant leaching occurred in the consecutive FADH reactions. Post-characterization after 15 cycles was performed using SEM, XRD, and XPS spectroscopy. The SEM photographs of PdAu@ACTNSs-1 taken after 5 and 15 cycles did not show any significant change in particle size or surface morphology (Figure S15 of the Supporting Information). The XRD patterns of as-synthesized and used PdAu@ACTNSs-1 after 5 and 15 consecutive runs were compared to evaluate the long-term stability of the catalyst (Figure S16 of the Supporting Information). The results demonstrate the characteristic diffraction peak positions of the anatase-type crystalline structure of TiO_2_-based support, confirming that no structural phase change occurred during the cyclic tests. On the other hand, a noticeable decrease in the peak intensities is observed after the 15th cycle, which can be attributed to a slight loss of crystallinity after prolonged catalytic tests. The overall structural framework of the anatase-phase support is well preserved. No significant peak shift or the appearance of a new peak with respect to the core-level spectra for Ti 2p, Pd 3d, Au 4f, O 1s, and C 1s were observed in the core-level XPS spectra of PdAu@ACNSs-1 after 5 and 15 consecutive runs (Figures S17 and S18 of the Supporting Information). The Ti­(III)/Ti­(IV) ratio and oxygen vacancy of the used catalyst (15 cycles of the thermocatalytic FADH reaction) were calculated to be 0.403 and 40.2%, respectively. These values are very close to those of the original catalyst (i.e., 0.409 and 40.7%). These analyses demonstrated that the active site content, the morphology, the crystalline structure, and the surface chemistry of PdAu@ACTNSs-1 were maintained during the consecutive thermocatalytic FADH runs performed at 60 °C.

The general reaction mechanism for the thermocatalytic dehydrogenation of formic acid on heterogeneous catalysts involves three main steps (Figure 8). First, the reaction predominantly follows the formate pathway. Formic acid (FA) is a weak acid with limited dissociation. The addition of sodium formate (SF) plays a crucial mechanistic role by serving as an abundant and direct source of formate ions (HCOO^–^). Furthermore, the equimolar (1/1) FA/SF mixture establishes an effective buffer system that facilitates continuous deprotonation of FA. These formate ions readily adsorb onto the catalyst surface, where the Pd sites serve as the primary adsorption centers to form a Pd-formate intermediate.^29,63^ The Au atoms in the nanoalloy modify the electronic structure of Pd, weakening the Pd–H bond and facilitating the second step, which is the cleavage of the C–H bond within formate.^29,30^ This leads to the third step of the reaction, the release of CO_2_, and the surface H atoms recombine to evolve H_2_ gas.^63^ Defects in the TiO_2_ crystal structure facilitate electron transfer and form more electron-rich PdAu nanoalloy surfaces, which increase the thermocatalytic performance.^56^ The glycolate-based structure of the TiO_2_ NSs synthesized in this work allowed the formation of amorphous regions, including carbon at considerably higher concentrations within the crystalline anatase matrix than those of TiO_2_ nanoparticles synthesized by other current protocols. The higher carbon concentration and higher oxygen vacancy of PdAu@ACTNSs-1 are the factors involved in the formation of more electron-rich PdAu nanoalloy surfaces within the anatase matrix with higher catalytic performance. The measured activation energy (45.2 kJ mol^–1^) and activation enthalpy (42.5 kJ mol^–1^) support a relatively low-barrier pathway, consistent with formate decomposition as the rate-determining step.^78^ The large negative entropy of activation (−108.8 J mol^–1^K^–1^) further confirms a transition state with high ordering, as expected for surface-anchored formate intermediates.^30,79^ The absence of CO in the GC–MS analysis indicates that the undesired dehydration pathway is effectively suppressed, most likely due to the synergistic Pd–Au interface, the optimized FA/SF system that prevents highly acidic conditions, and the defect-rich carbon-decorated anatase TiO_2_ support, which promotes proton transfer and electron delocalization.^29,30^

**Figure 8:**
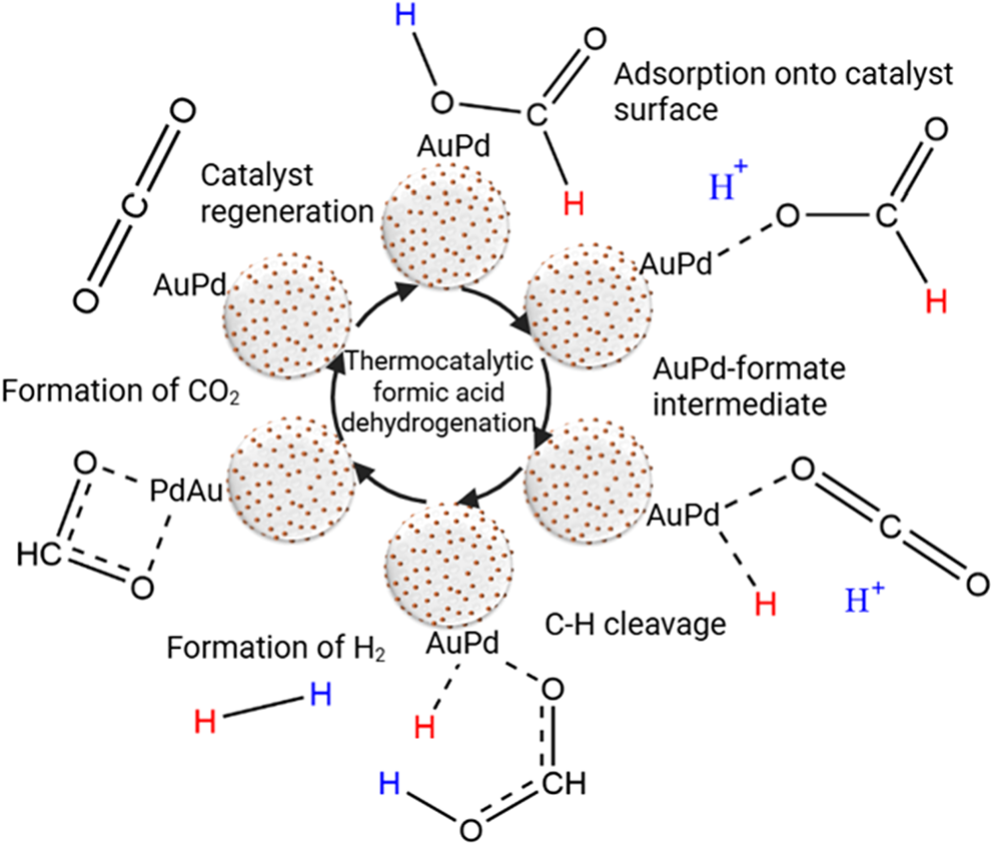
Schematic representation of the reaction mechanism in thermocatalytic FADH.

#### 3.3.2 Photocatalytic FADH Experiments

The catalyst providing the best performance in thermocatalytic FADH reaction (i.e., PdAu@ACTNSs-1) was also evaluated in the photocatalytic FADH runs performed at 25 °C. The reaction was initiated using a visible-light source, which was set 15 cm away from the reactor to provide 1.0 sun power. The most appropriate reaction conditions determined in the thermocatalytic FADH runs were also used for these runs. The kinetic curves obtained using different catalyst concentrations in the photocatalytic FADH reaction are shown in Figure 9A. The TOF values are presented in Table S3 of the Supporting Information. The results show that the appropriate concentration for the photocatalytic FADH reaction is 30 mg mL^–1^, which will be used for further catalytic runs. Note that the photocatalytic FADH reaction at 25 °C was slower than the thermocatalytic FADHs performed at higher temperatures. Depending on this behavior, lower TOF values than those of thermocatalytic FADH runs were obtained. The highest TOF in the photocatalytic experiments was 1507.7 h^–1^ with a catalyst concentration of 30 mg mL^–1^ at 25 °C. This value was higher than those obtained with similar current photocatalysts used in visible-light-driven photocatalytic FADH runs (Table S3 of the Supporting Information).

**Figure 9:**
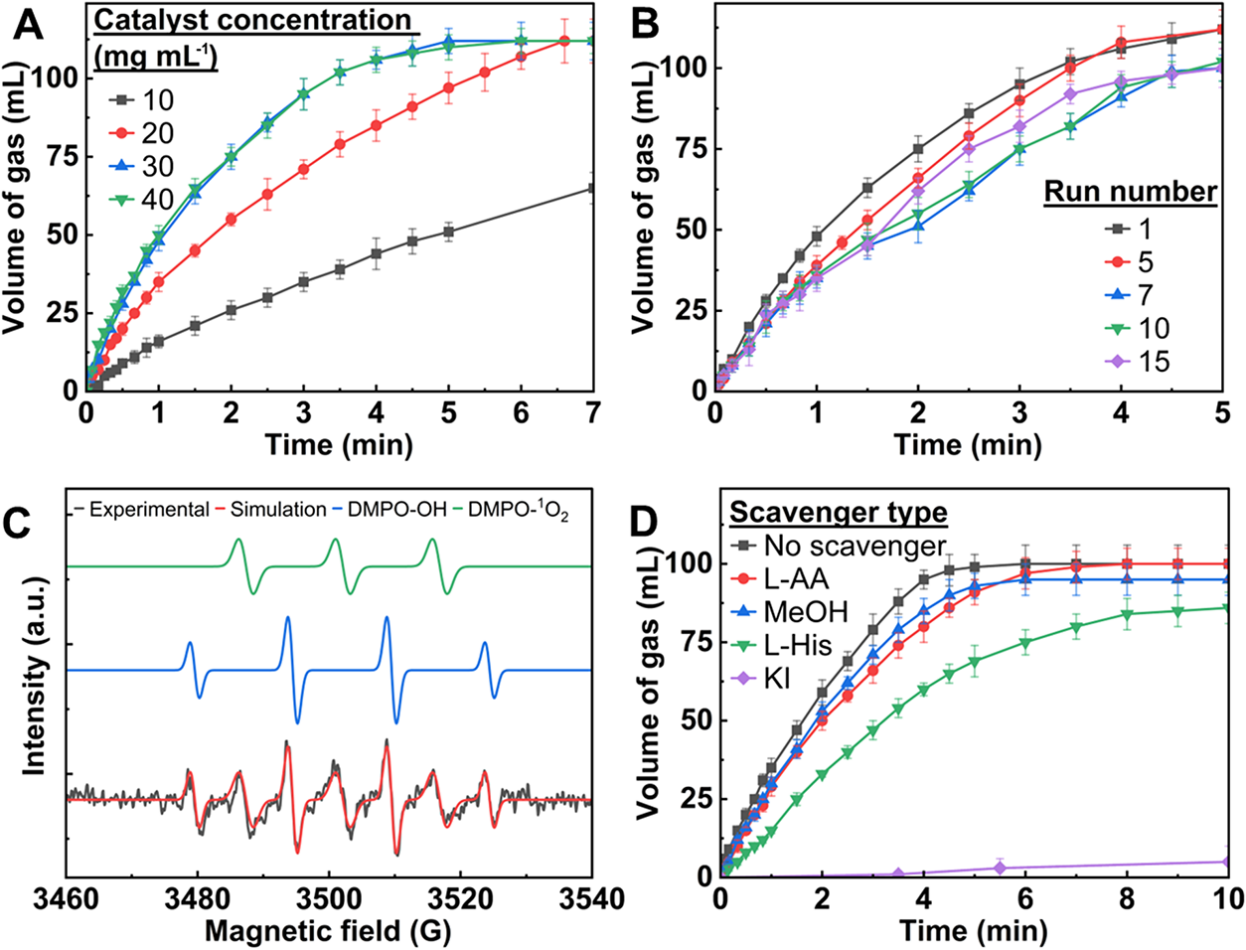
(A) Effect of PdAu@ACTNSs-1 concentration, (B) reusability of PdAu@ACTNSs-1 in the visible-light-driven photocatalytic FADH reaction. Conditions (unless otherwise stated): Pd/Au weight ratio: 1/1, PdAu loading: 5.0 wt %/wt., FA/SF mole ratio: 1/1, PdAu@ACTNSs-1 concentration: 30 mg mL^–1^, temperature: 25 °C, stirring rate: 350 rpm under visible light. (C) Experimental and simulated ESR spectra obtained using PdAu@ACTNSs-1 under visible-light irradiation, and (D) detection of possible radicals by including different radical scavengers in the FADH reaction medium.

For photocatalytic FADH, the reusability runs are presented in Figures 9B and S19 of the Supporting Information. The catalyst exhibited >90% conversion in all the runs. Although a slightly lower conversion rate was observed in the third run, the kinetic curves remained almost the same even after 15 cycles. The observed drop in the conversion could be attributed to the temporary accumulation of the chemical species on the surface of the catalyst during the intermediate cycles. However, the recovery of >90% conversion in the final cycle suggests that the catalyst retains good stability and sustained catalytic activity even after 15 consecutive cycles.

ESR analysis was conducted to determine radical generation in the photocatalytic FADH reaction. The analysis detected the generation of reactive oxygen species by the catalyst PdAu@ACTNSs-1. The ESR spectra are shown in Figure 9C. In order to obtain the ESR parameters of the radical species, a simulation was conducted in MATLAB utilizing the nonlinear least-squares solver fminsearch. The simulation revealed the presence of ^•^OH and ^1^O_2_ radicals under the effect of visible light by PdAu@ACTNSs-1 in the form of DMPO adducts. The sextet signal related to the DMPO–OH adduct had the hyperfine splitting constants of *A*_N_ = 15.04 G and *A*_H_ = 14.75 with a g-factor of 2.0057 and intensity ratios of 1:2:2:1. The signals with 1:1:1 intensity ratios were ascribed to the DMPO–^1^O_2_ adduct with a hyperfine splitting constant of *A*_N_ = 14.73 and a g-factor of 2.0056.

A radical scavenging experiment to detect the generation of reactive oxygen species and electron holes under visible light was conducted utilizing L-AA, MeOH, l-His, and KI as scavengers for O_2_^–^•^^, ^•^OH, ^1^O_2_, and holes, respectively. The catalytic behavior in the presence of different scavengers is shown in Figure 9D. It was observed that when L-AA was used as the scavenger, the FADH rate slightly decreased, but the reaction system eventually reached the expected gas volume, indicating that no significant O_2_^–^•^^ radical generation occurred during the photocatalytic experiment. The MeOH experiment presented a slight decrease in the catalytic behavior and did not achieve full conversion of FA, which indicated the formation of a small amount of ^•^OH during the experiment. On the other hand, the addition of l-His to the reaction medium significantly influenced the catalytic behavior, indicating the generation of ^1^O_2_ species during the photocatalytic FADH. Electron holes were detected clearly using KI as the chemical scavenger during FADH under visible light. This chemical could also be used for the clear detection of ^•^OH.^79^ These results are consistent with the ESR analysis results, indicating the generation of ^1^O_2_ and ^•^OH radicals.

After conducting the FADH reaction, a sample of the evolved gas was preserved in a special gas vial and then introduced into a GC-MS analyzer to detect the presence of CO in the evolved gas from the reactor. Figure S20 of the Supporting Information shows the GC-MS analysis, where the presence of a dominant peak at *m*/*z* 44.1 confirms the presence of CO_2_ as the major ingredient in the sample. Minor signals at *m*/*z* 12.1 and 16.1 are attributed to the fragmentation products of CO_2_ (i.e., fragment ions of carbon and oxygen, respectively). Most importantly, the absence of a strong signal at *m*/*z* of 28 indicated that no significant amount of CO was detected during the reaction and confirmed 100% H_2_ selectivity toward the photocatalytic FADH reaction.

Under visible-light irradiation, the reaction mechanism involves both charge-carrier processes from carbon-decorated TiO_2_ and the interfacial catalytic role of the PdAu nanoalloy (Figure 10).^30,80^ The incorporation of C atoms in the crystal structure extends the visible-light absorption of TiO_2_ and promotes the generation of electron–hole pairs.^80^ Because of the work function differences between the semiconductor and noble metals, a Schottky barrier is formed at the metal–support interface.^81^ This junction allows the PdAu nanoalloy to act as a highly efficient electron sink. Consequently, photogenerated electrons (e^–^) rapidly migrate from the conduction band of carbon-decorated TiO_2_ to the PdAu surface. This directional and spatial charge separation effectively prevents the recombination of e^–^/h^+^ pairs, significantly prolonging the lifetime of the charge carriers.^81^

**Figure 10:**
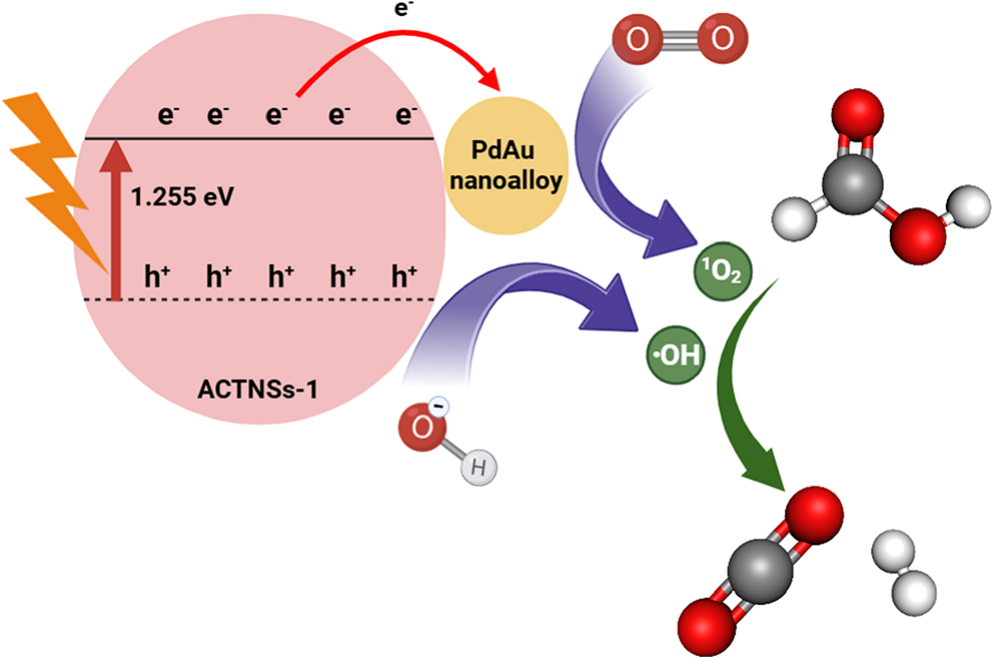
Photocatalytic reaction pathway diagram for PdAu@ACTNSs-1.

Scavenger experiments revealed that photogenerated holes (h^+^) are directly involved in the oxidation of formate intermediates, as confirmed by the significant inhibition observed with KI.^30^ In addition, singlet oxygen (^1^O_2_) was detected as the main reactive oxygen species, with smaller contributions from hydroxyl radicals (^•^OH). The radical oxygen species formed during the photocatalytic dehydrogenation reaction facilitate the oxidative decomposition of FA and its intermediates, and the formation of CO_2_. Meanwhile, photogenerated electrons are efficiently trapped at the PdAu alloy sites, where they combine with protons released during FA oxidation to produce molecular hydrogen.^30,82–84^ The cooperative effect between ROS-driven oxidation and metal-assisted proton reduction explains the high photocatalytic activity and selective evolution of H_2_ and CO_2_ without detectable CO.^30,82,84^

## 4 Conclusion

The present research focused on developing titanium glycolate-based supports for the immobilization of active sites to synthesize a catalyst with high catalytic performance for the decomposition of FA into H_2_ and CO_2_ without producing CO as confirmed by GC-MS analysis. Among all, the catalyst PdAu@ACTNSs-1 exhibited the best catalytic activity for the dehydrogenation of FA. The high catalytic performance is attributed to the significant structure of the catalyst supported by anatase nanospheres decorated with carbon, which have relatively low crystallinity and low crystallite size obtained by controlling the hydrolysis conditions. Tunable hydrolysis also provided a high Ti­(III)/Ti­(IV) ratio and high oxygen vacancy concentration in the resulting catalyst. Based on these properties, the PdAu@ACTNSs-1 catalyst functions efficiently as both a thermocatalyst and a photocatalyst, with outstanding performance and exceptional TOF values of 20,924 h^–1^ and 1507.7 h^–1^, respectively, which have not been observed for the similar current catalysts. This novel approach can be evaluated for the synthesis of heterogeneous catalysts/photocatalysts for various organic reactions other than the catalytic dehydrogenation of FA. The control of the crystalline properties, carbon content, oxygen vacancy, and Ti­(III)/Ti­(IV) ratio of TiO_2_-based catalysts only by adjusting the hydrolysis period of the support is a novel finding that is unique to titanium glycolate-based nanomaterials, discovered in this work. Therefore, high-performance catalysts that are able to provide exceptional TOF values can be potentially synthesized using the titanium glycolate-derived supports by adopting the synthetic route established in this work for FADH catalysts. The catalyst PdAu@ACTNSs-1 also presents high stability and durability by maintaining the same size, crystallographic, and surface chemical properties even after the implementation of consecutive catalytic cycles with the same superior catalytic performance, as confirmed by SEM, XRD, and XPS. The catalyst PdAu@ACTNSs-1 generates ^1^O_2_, ^•^OH radicals, and electron holes during the photocatalytic FADH reaction, which were detected by ESR analysis and chemical scavengers. The reusability of the PdAu@ACTNSs-1 catalyst was confirmed by recyclability runs for both thermo- and photocatalytic FADH, where the catalyst maintained superior performance after five catalytic runs.

## Supplementary Material



## Data Availability

The data that support the findings of this study are available in the Supporting Information of this article.

## Notes

Additional experimental details, materials, and methods, including post-characterization of the catalyst after recycling (PDF)
