# Supplementary material for: Hydrogen Evolution Performance of a PdAu-Loaded Titanium Glycolate-Based Catalyst in Formic Acid Dehydrogenation with a High Turnover Frequency
Source: ACS Omega. 2026 May 26;11(23):34357–72. doi: 10.1021/acsomega.6c02175 (PMC13280994; doi:10.1021/acsomega.6c02175)
Supplement: Supplementary file 1 [file ao6c02175_si_001.pdf]

Hydrogen evolution performance of a PdAu loaded titanium glycolate based catalyst in formic acid dehydrogenation with a high turnover frequency

Razan Anwar Hamdan[1], Duygu Hacıefendioğlu[1], Burcu Gökçal Kapucu[1],

Mustafa Polat[2], Ali Tuncel[1,a]

[1] Hacettepe University, Chemical Engineering Department, Ankara, Turkey

[2] Hacettepe University, Department of Physics Engineering, Ankara, Turkey

a: Corresponding author: Dr. Ali Tuncel, Hacettepe University, Chemical Engineering Department, 06800, Ankara, Turkey (atuncel@hacettepe.edu.tr)

Supporting Information

S1. Experimental

S1.1. Materials

For the synthesis of TGNSs, titanium (IV) isopropoxide (97%, Sigma Aldrich Chem Corp, St. Louis, Missourri, ABD) and ethylene glycol (Emplura, Merck AG, Darmstad, Germany) were used as the titanium precursor and the glycolate precursor, respectively. Acetone (Emsure, Merck) and technical ethanol (96%) was used without further purification. Deionized (DI) (18 MΩ.cm) water obtained using Direct-Q3, Millipore, U.S.A. 3-(aminopropyl) triethoxysilane (APTES, Sigma Aldrich), triethylamine (TEA, Sigma Aldrich), and 2-propanol (Emsure, Merck) were used in the amine functionalization of nanospheres. The metal loading experiments were conducted using polyvinyl alcohol (PVA, 87.000-146.000 Da, 87 % hydrolysed, Sigma Aldrich) as a stabilizer, gold (III) chloride trihydrate (Sigma Aldrich) and palladium (II) nitrate dihydrate (Sigma Aldrich) as the precursors, and sodium borohydride (Sigma Aldrich) as the reductant. The catalytic runs were performed with formic acid (FA, Sigma Aldrich) and sodium formate (SF, Fisher). For the determination of radicals generated by the catalysts, 5,5-dimethyl-1-pyrroline N-oxide (DMPO, Sigma Aldrich) was selected as the spin-trapping agent in the ESR experiments. L-ascorbic acid (L-AA, Sigma Aldrich), L-histidine (L-His, Merck), methanol (MeOH, J.T. Baker), and potassium iodide (KI, Sigma Aldrich) were used as the inhibitors in the aqueous medium.

S1.2. Functionalization of TGNSs, CTNSs by APTES

TGNSs, CTNSs-1, CTNSs-2 or CTNSs-3 (100 mg) were dispersed in isopropanol (10 mL) by ultrasonication sonication and vortexing for 5 min. The dispersion was transferred to a glass T-shaped sealed reactor. Then, APTES (1 mL) was added to the reaction medium, followed by TEA (0.15 mL) as the catalyst. The reactor was kept at 80°C with continuous shaking at 120 cpm, overnight. APTES attached-nanospheres, obtained from TGNSs, CTNSs-1, CTNSs-2 and CTNSs-3, referred as ATGNSs and ACTNSs-1, ACTNSs-2 and ACTNSs-3, respectively were washed with isopropanol and DI water twice by a successive centrifugation and decantation protocol and dried in vacuum at 60°C.

S1.3. The syntheses of heterogeneous catalysts by loading of PdAu ultrafine nanoalloy on ATGNSs and ACTNSs.

TGNSs and CTNSs with different crystalline properties were used as supports for the preparation of FADH catalysts. The feed ratio of ultrafine PdAu alloy with respect to TiO_2 based support was selected as 5.0 wt. %. The feed ratio of 2.5 wt.% was used for both Pd and Au loadings. For comparison, the catalysts were also prepared with different Pd/ Au feed ratios by keeping the total ratio of PdAu alloy to TiO_2 as 5.0 wt. %. The metal immobilization over the amine-treated supports was performed by in-situ precipitation. PVA (12 mg) was added to DI water (15 mL), and the medium was subjected to magnetic stirring at 350 rpm for 24 h for dissolution of PVA. Then, the

selected support (200 mg) was finely dispersed in DI water (3 mL) by ultrasonication for 1 min and the obtained dispersion was added to the PVA solution. Pd precursor (palladium (II) nitrate dehydrate, 12.5 mg) and the gold precursor (gold (III) chloride trihydrate, 10.0 mg) were separately dissolved in DI water (1 mL). They were added to aqueous dispersion containing PVA and the support NPs. The dispersion was stirred for 1 h at room temperature. $NaBH_4$ solution (0.1 M, 3.6 mL) was added to the reaction medium to initiate the reduction process and the transformation of the metal ions into metal nanoparticles immobilized on the surface of amine-functionalized support. The reaction was set to proceed for 1 h after the addition of $NaBH_4$. The catalyst was collected and washed with DI water and dried in vacuum oven at 60 °C overnight. The catalysts obtained by loading PdAu NPs onto titanium glycolate based supports with different crystalline properties were referred as PdAu@ATGNSs, PdAu@ACTNSs-1, PdAu@ACTNSs-2, PdAu@ACTNSs-3.

S1.4. Characterization of catalysts

The surface morphology and size distribution characteristics were investigated for both bare TGNSs, CTNSs and their PdAu bimetallic alloy loaded forms using a Scanning Electron Microscope (SEM, Tescan, Czech Republic). For this purpose, a small amount of dried nanospheres was dispersed perfectly in an aqueous sodium dodecyl sulfate solution by ultrasonication, dropped onto carbon tape, and coated with gold-palladium in a sputter coater (Polaron, Range sputter coater, UK) for imaging. Specific surface area (SSA), pore volume and

pore size distribution were determined by N_2 adsorption/desorption method, according to Brunauer–Emmett–Teller (BET) model using a pore size and surface area analyzer (Nova 2200 E, Quantachrome, UK). Prior to the BET measurements, the samples were degassed under vacuum at 120 °C for 6 h. X-ray diffraction (XRD, Rigaku MiniFlex, Japan) analysis was utilized to investigate the crystallographic properties of TGNSs, CTNSs, PdAu@ATGNSs, and PdAu@ACTNSs. The measurements were conducted using Cu Kα radiation at an operating voltage of 40 kV and a current of 15 mA. The diffraction patterns were recorded in the 2θ range of 2° to 80° with a step size of 0.02° and a scanning speed of 5.00°/min. The surface chemical properties of the catalysts were analyzed using an X-ray Photoelectron Spectrometer (XPS, K-Alpha, Thermo Fisher Scientific, USA) equipped with a monochromatic Al Kα X-ray source (1486.7 eV). The binding energy scale of the instrument was initially calibrated against the C 1s peak at 284.8 eV using polyethylene terephthalate (PET) as a reference material. Therefore, no further binding energy calibration was applied to the obtained spectra during data processing. Inductively Coupled Plasma Optical Emission Spectroscopy (ICP-OES) (Perkin Elmer DRC II, U.S.A.) analysis was utilized to investigate the Pd and Au contents of PdAu@ TGNSs, and PdAu@ACTNSs. Before the ICP-OES analysis, the solid powder samples were completely digested in aqua regia. The hydrodynamic size distributions in DI water and also FADH reaction medium were determined by Dynamic Light Scattering (DLS, Malvern Instruments, U.K.). Fourier Transform Infrared (FTIR) spectra of the samples were recorded using a spectrometer

(Thermoscientific Nicolet 6700 U.S.A.) equipped with an Attenuated Total Reflectance (ATR) accessory featuring a diamond crystal. The measurements were performed directly on the solid powder samples without any KBr dilution. All spectra were collected in the wavenumber range of 4000 to 400 cm^{-1} with a spectral resolution of 4 cm^{-1}. For each measurement, 32 scans were co-added to ensure a high signal-to-noise ratio. The quantitative elemental composition (Carbon, Hydrogen, Nitrogen, and Sulfur) of the samples was determined using an Elemental Analyzer (LECO TruSpec Micro Elemental Analyzer, U.S.A). Briefly, 2 mg of the dried catalyst sample was weighed into a tin capsule. The samples were subjected to combustion in a furnace at 950 ^{o}C under an oxygen atmosphere, and helium as the carrier gas. To eliminate excess oxygen a copper column was used. The instrument was calibrated using EDTA and sulfamethazine as standards. To ensure high accuracy and reproducibility, consecutive measurements were performed for each sample, and the elemental contents were reported as average weight percentages (wt%). The detection of CO formation in the product of FADH reaction was performed using Gas Chromatography-Mass Spectroscopy (GC-MS, Agilent Technology, 7890B, CA, U.S.A.). UV-visible spectrophotometer (Thermoscientific, U.S.A.) was utilized to obtain the Tauc plot and to determine the band gap energy of PdAu@ACTNSs. For these optical measurements, the solid samples were uniformly dispersed in deionized water. For photocatalytic FADH experiments, a white COB LED (CITIZEN, 48.7 W, 4000K, Japan) was used as the visible light source. Electron Spin Resonance Spectrometer (ESR, Bruker EMX131 X-Band, Germany) was utilized for the detection of radicals generated by PdAu@ACTNSs under visible light irradiation, and to identify

the oxygen vacancies within the catalysts. To detect the oxygen vacancies, ESR spectra of the dry powder samples were recorded at 290 K. The instrumental parameters for the oxygen vacancy analysis were set as following microwave frequency: 9.771 GHz, microwave power: 1 mW, modulation frequency: 100 kHz, modulation amplitude: 2 G, and sweep resolution: 1024 points. The g-values corresponding to the trapped electrons were calculated from the applied microwave frequency and the magnetic field.

S1.5. Thermocatalytic formic acid dehydrogenation

TOF values regarding to each thermocatalytic FADH reaction was calculated with Equation S1.

$$TOF = \frac{PV}{2RTn_{active\ site}t_{\%20}} \qquad \text{(S1)}$$

In Equation S1, P is the pressure of the gas collected in the cylinder (1 atm), V is the volume of the gas obtained at 20% FA conversion, R is the ideal gas constant, T is the temperature. n_{active} site is the total mole of Pd and Au metals calculated from ICP-OES results and $t_{20\%}$ is the time required to reach 20% conversion of FA.

To evaluate the stability and reusability of the catalyst, consecutive formic acid dehydrogenation (FADH) runs were performed under identical experimental conditions. After the completion of each catalytic run, the solid catalyst was successfully recovered from the reaction mixture via centrifugation. The recovered catalyst was then washed thoroughly with deionized water several

times to remove any residual unreacted formic acid and organic byproducts. Subsequently, the washed catalyst was directly used for the next consecutive run without requiring any further thermal or chemical regeneration treatments.

S1.6. Photocatalytic formic acid dehydrogenation

TOF values for FADH runs were calculated using the equation given in Section S1.8 for the thermocatalytic runs. Given that vacancy-rich titania can potentially induce local temperature increases under illumination due to photothermal effects,[S1] the temperature during the photocatalytic experiments was monitored using a thermocouple. To minimize any thermal contribution to the catalytic activity, a dual-cooling system was employed. The light source was equipped with an integrated cooling fan, and the reactor was placed in a cabinet with a thermostat-connected cooling fan. Throughout the illumination period, the reaction temperature was successfully maintained at 25 ± 2 ˚C. The reusability tests for the photocatalytic experiments were conducted following the exact same catalyst recovery and washing procedures described for the thermocatalytic runs.

S2. Synthesis protocols of catalysts

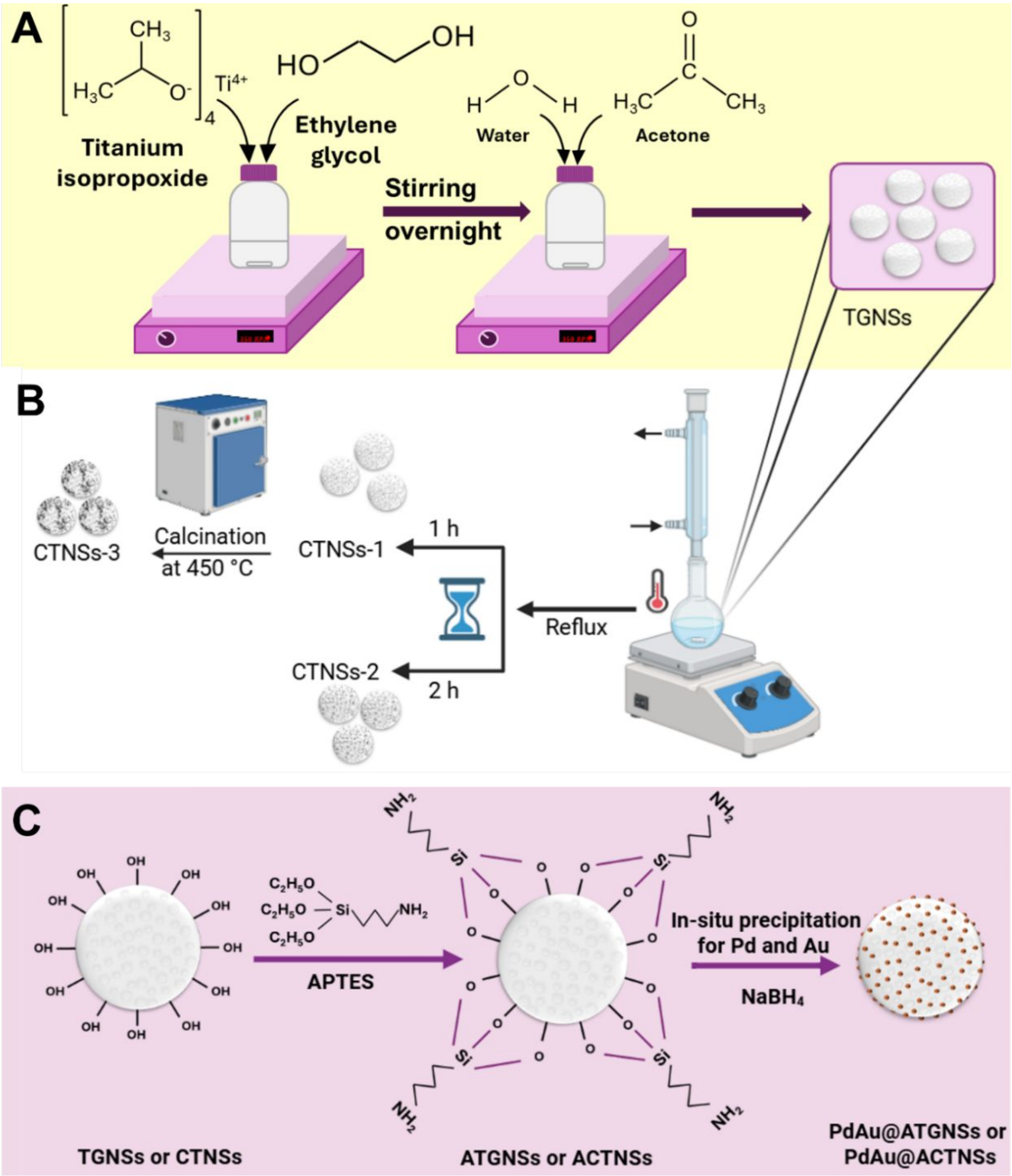

Figure S1. The synthetic pathways for obtaining titanium glycolate based NSs and their usage as supports in the synthesis of heterogeneous catalysts via $NaBH_4$ reduction of Pd and Au salts. (A) Synthesis of TGNSs, (B) Synthesis of carbon doped mesoporous TiO_2 NSs via refluxing for 1 h

and 2 h and also refluxing for 1 h and calcination at 450 °C (C) Attachment of PdAu nanoalloy onto titanium glycolate based nanospheres.

S3. Porous properties of titanium glycolate based nanospheres

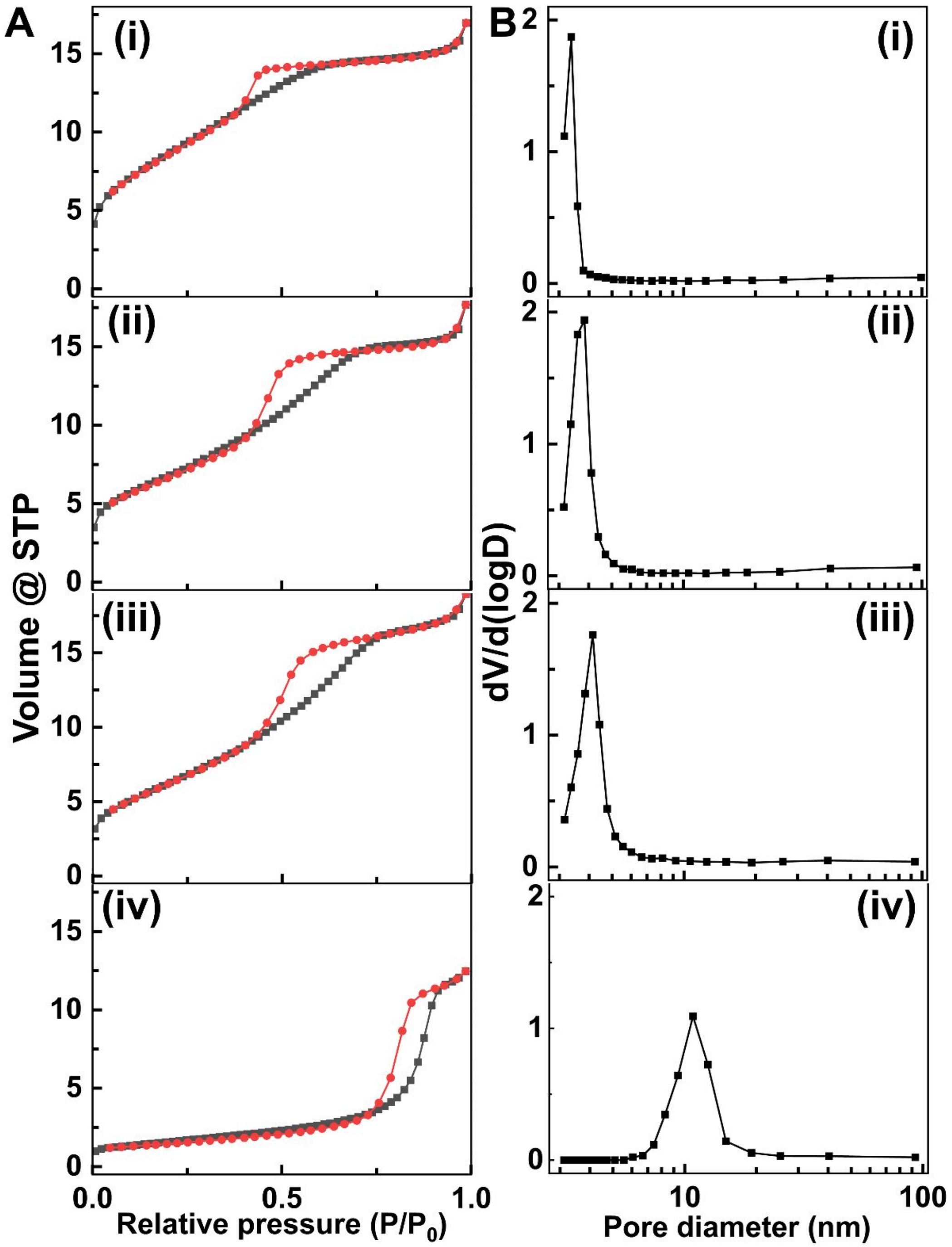

Figure S2. (A) N_2 adsorption/desorption isotherms and (B) Pore size distributions of (i) TGNSs, (ii) CTNSs-1 (reflux time: 1 h), (iii) CTNSs-2 (reflux time: 2 h), and (iv) CTNSs-3 with a reflux period of 1 h and calcination at 450 °C.

Table S1. Porous properties of different titanium glycolate based nanospheres.

Support	SSA (m^2 g^{-1})	Pore volume (cm^3 g^{-1})	Average pore diameter (nm)
TGNSs	333	0.159	3.4
CTNSs-1	268	0.257	3.8
CTNSs-2	236	0.270	4.1
CTNSs-3	60	0.218	10.8

TGNSs: Mesoporous titanium glycolate nanospheres, CTNSs-1: Mesoporous anatase TiO_2 nanospheres (reflux time: 1 h), CTNSs-2: Mesoporous anatase TiO_2 nanospheres (reflux time: 2 h), CTNSs-3: Mesoporous anatase TiO_2 nanospheres produced with a reflux time of 1 h and then calcination at 450 °C for 4 h.

S4. FTIR spectra of titanium glycolate based nanospheres

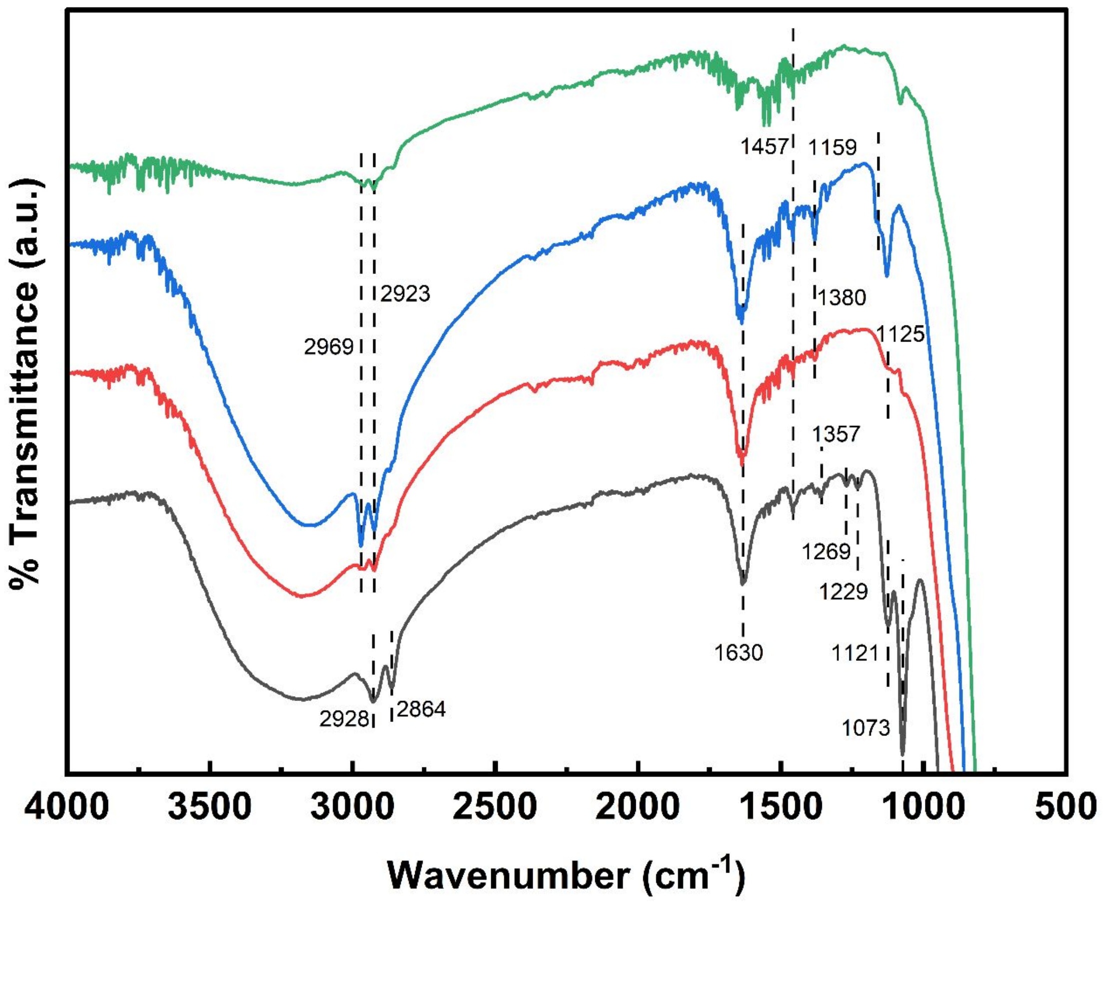

Figure S3. FTIR spectra for TGNSs (black line), CTNSs-1 (red line), CTNSs-2 (blue line), and CTNSs-3 (green line).

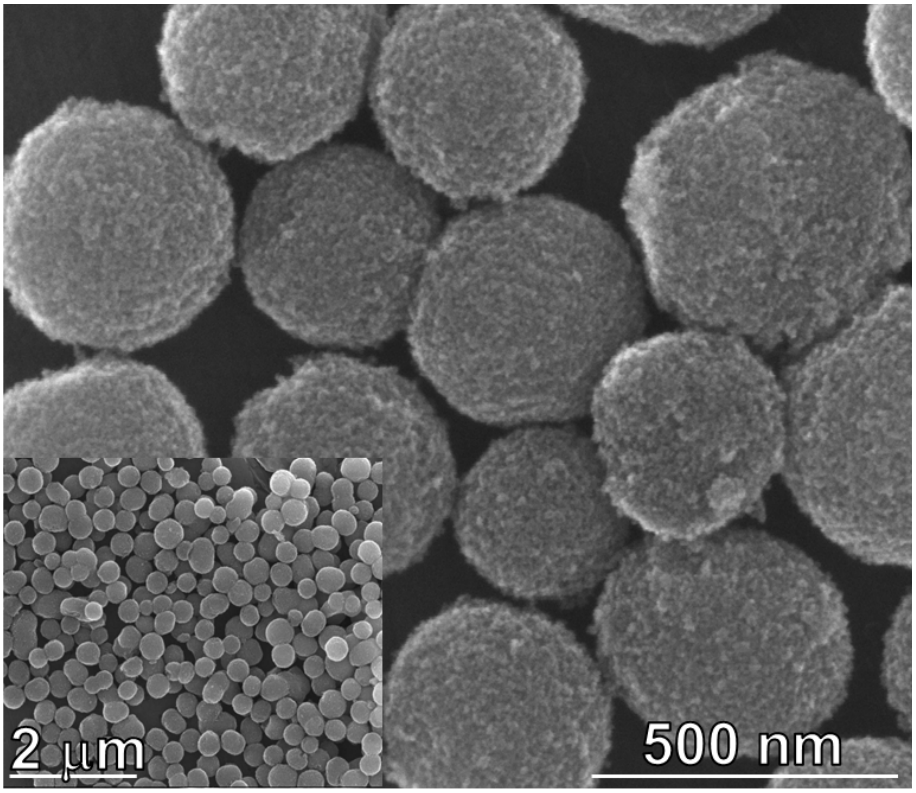

Figure S4. SEM images of plain CTNSs-1.

S5. TEM images and EDAX spectra of PdAu@ACTNSs-1

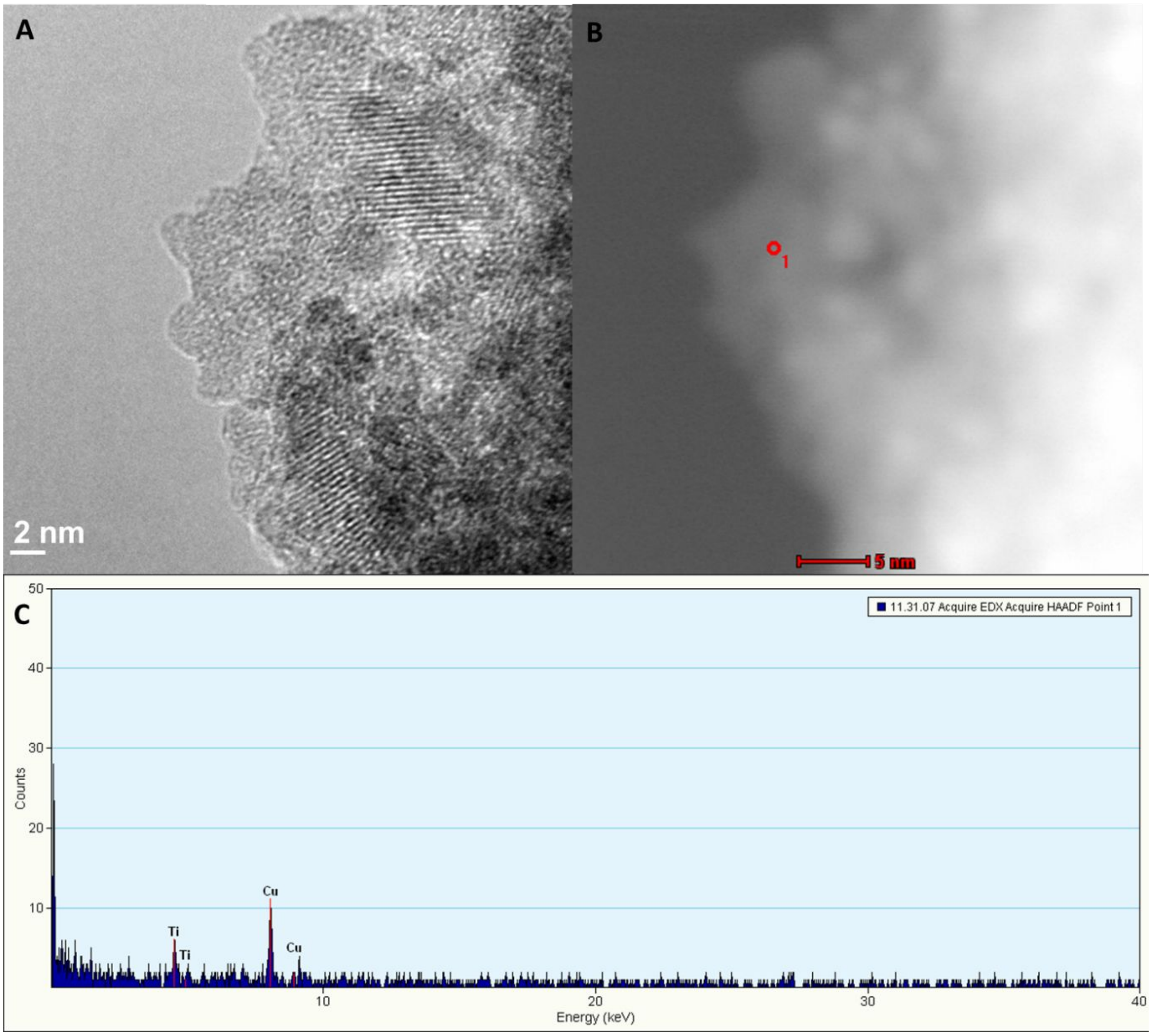

Figure S5. (A) TEM image of PdAu@ACTNSs-1, (B) HAADF image of the same area (red circle shows the EDAX point of the amorphous section), (C) EDAX spectrum of the amorphous section of PdAu@ACTNSs-1.

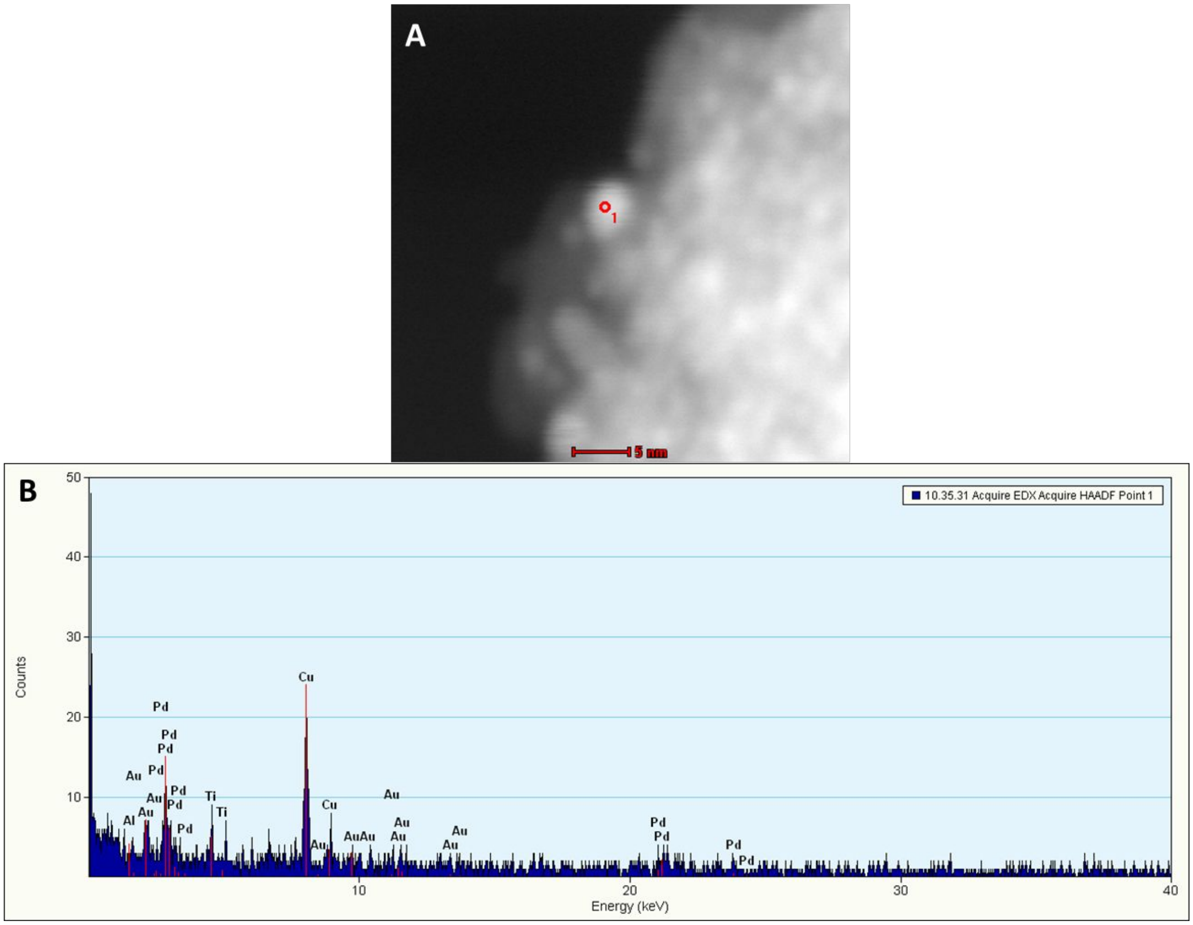

Figure S6. (A) HAADF image and (B) the corresponding EDAX spectrum of an individual PdAu nanoalloy on ACTNSs-1.

S6. Size distribution analysis

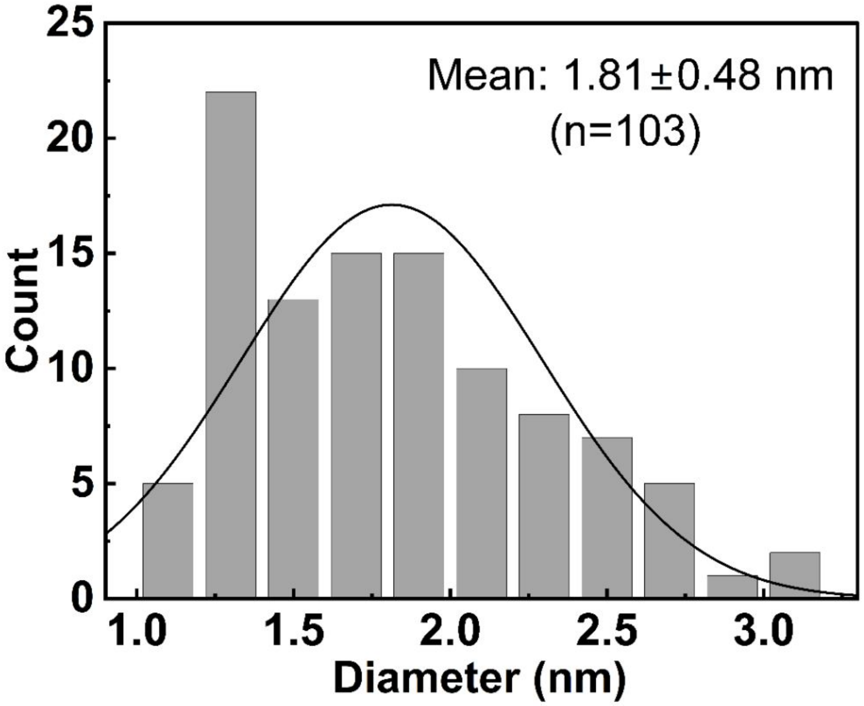

Figure S7. Histogram of the PdAu nanoalloy size distribution of PdAu@ACTNSs-1 calculated based on TEM images (i.e. Figure 2H)

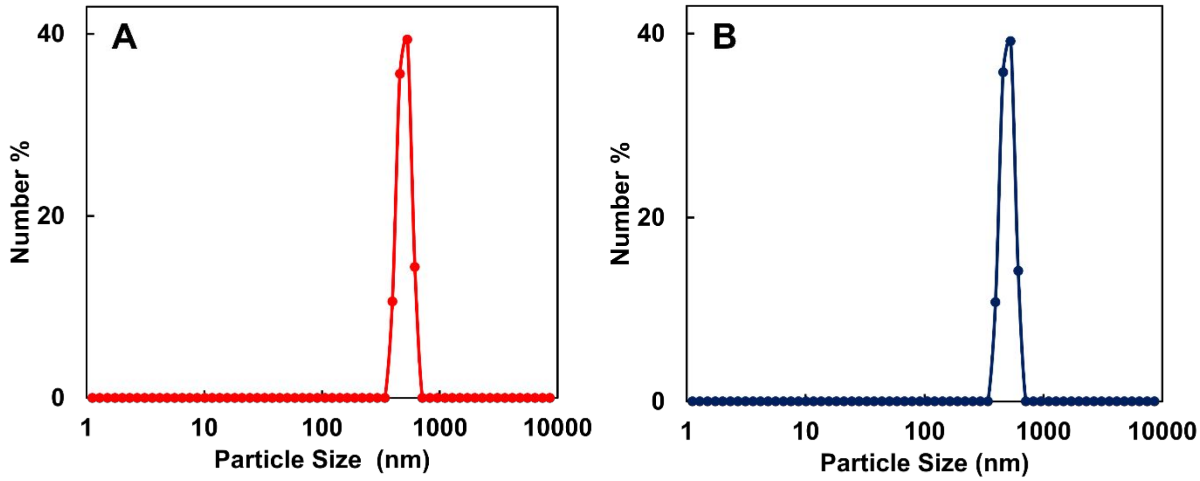

Figure S8. Hydrodynamic size distribution curve of PdAu@ACTNSs-1 in A) DI water, and B) in the FADH reaction medium containing 0.5 M FA and 0.5 M SF.

S7. Survey XPS and core level spectra for Ti 2p, O 1s and C 1s scan with titanium glycolate based nanospheres

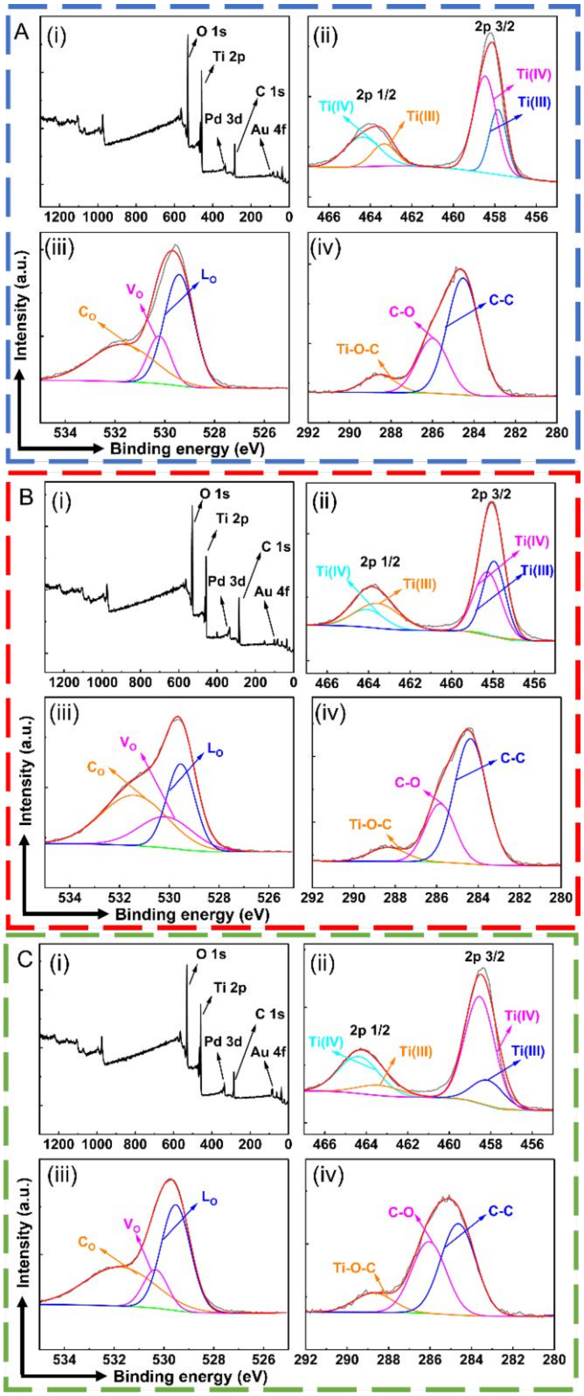

Figure S9. (i) Survey XPS spectra and core level spectra for (ii) Ti 2p, (iii) O 1s and (iv) C 1s scan with (A) PdAu@ATGNSs, (B) PdAu@ACTNSs-2 and (C) PdAu@ACTNSs-3.

S8. ESR analysis of Ti^{3+} species and oxygen vacancies

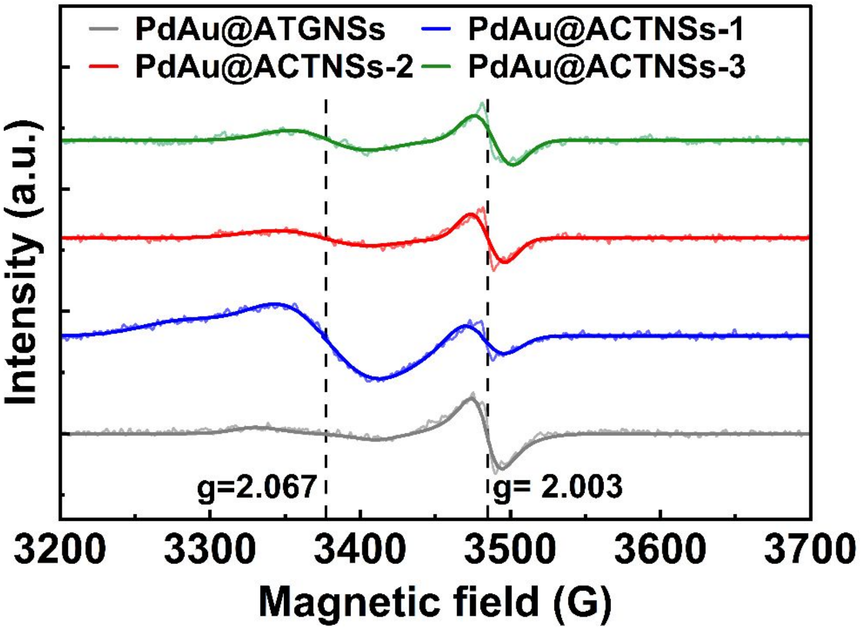

Figure S10. ESR spectra of PdAu@ATGNSs, PdAu@ACTNSs-1, PdAu@ACTNSs-2, and PdAu@ACTNSs-3

S9. Elemental analysis (CHNS) of the catalysts

Table S2. CHNS elemental analysis of different titanium glycolate based nanospheres.

Catalyst	H (%)	N (%)	S (%)
PdAu@ATGNSs	2.48	2.24	-
PdAu@ACTNSs-1	1.27	1.02	-
PdAu@ACTNSs-2	1.06	0.91	-
PdAu@ACTNSs-3	0.26	0.29	-

S10. Tauc plot

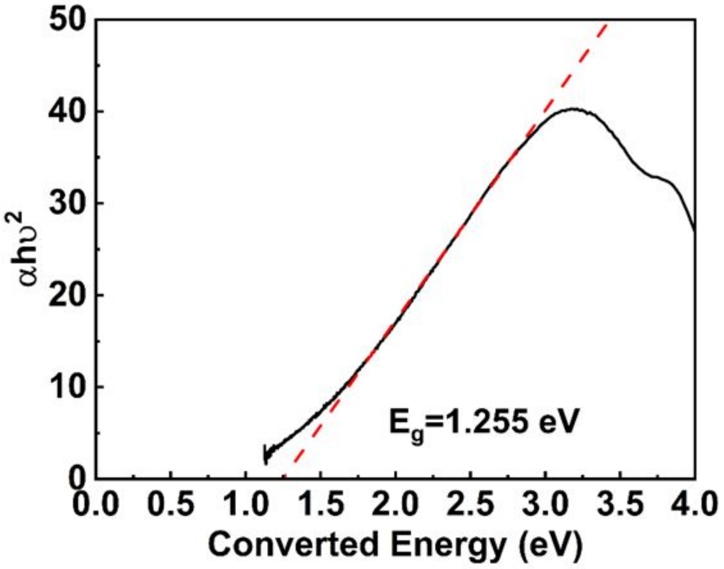

Figure S11. The Tauc plot of PdAu@ACTNSs-1.

S11. GC-MS spectrum of evolved gas sample in thermocatalytic FADH reaction

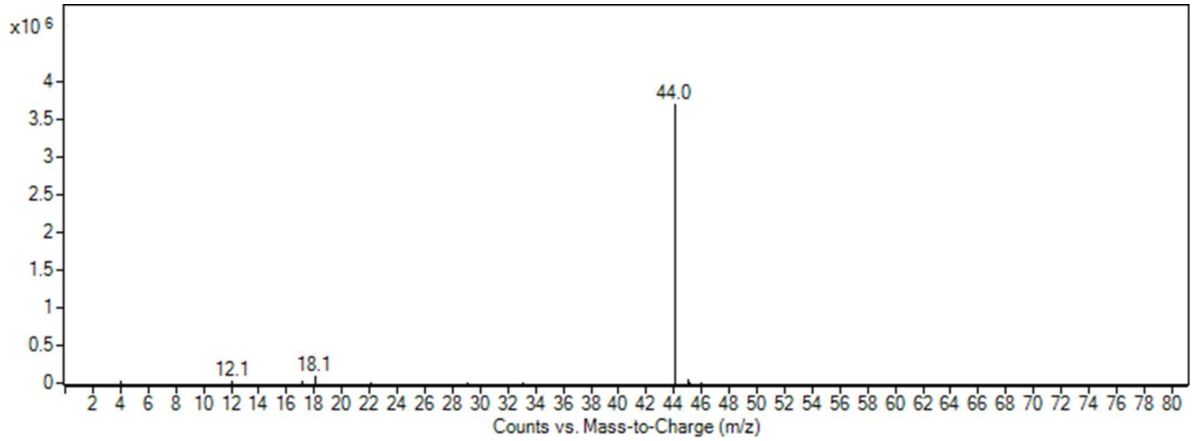

Figure S12. GC-MS spectrum of evolved gas sample in the thermocatalytic FADH reaction using PdAu@ACTNSs-1 at 60 ^{o}C. Conditions: Pd/Au weight ratio: 1/1, PdAu loading 5.0% wt./wt., FA/SF:1/1, PdAu@ACTNSs-1 concentration: 20 mg mL^{-1}, stirring rate: 350 rpm in dark.

S12. Arrhenius plot and Eyring plot for thermocatalytic FADH reaction with PdAu@ACTNSs-1

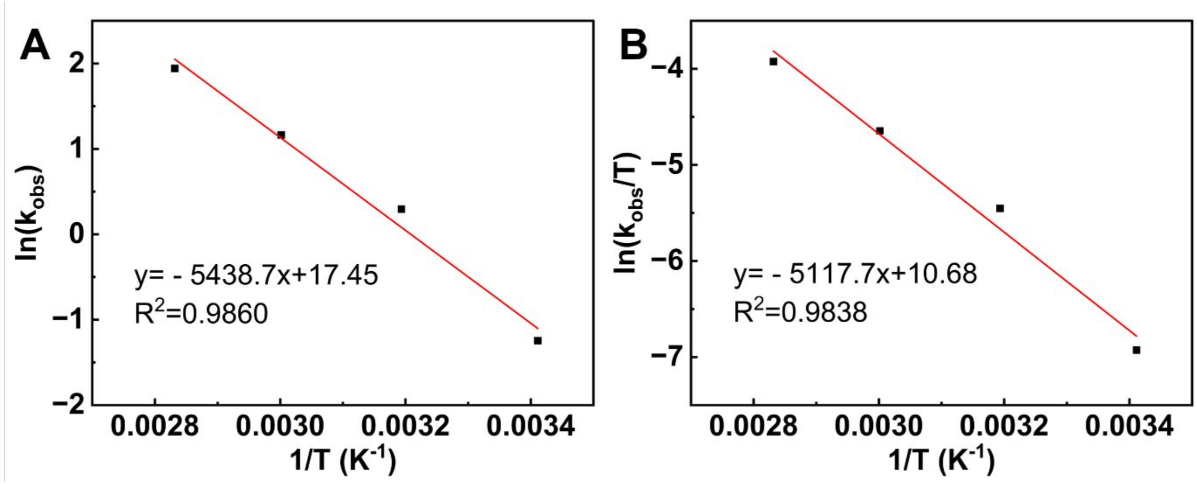

Figure S13. A) Arrhenius plot and B) Eyring plot constructed for thermocatalytic FADH reaction with PdAu@ACTNSs-1 as the catalyst.

S13. Reusability of PdAu@ACTNSs-1 in thermocatalytic FADH runs

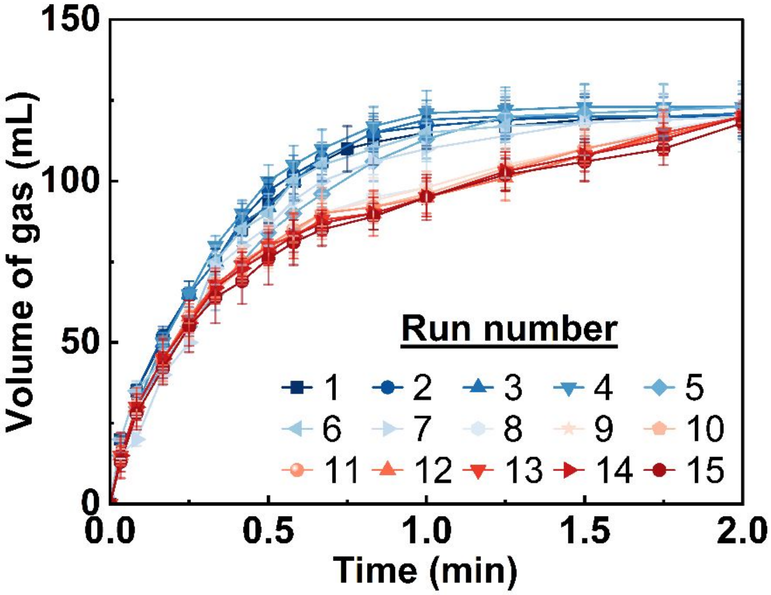

Figure S14. Reusability of PdAu@ACTNSs-1 in thermocatalytic FADH reaction. Conditions: catalyst concentration: 20 mg mL^{-1}, Pd/Au weight ratio: 1/1, PdAu loading 5.0% wt./wt., FA/SF mole ratio: 1/1, temperature: 60 ˚C, stirring: 350 rpm in dark.

S14. Post characterization of PdAu@ACTNSs-1 after recycling of thermocatalytic FADH runs

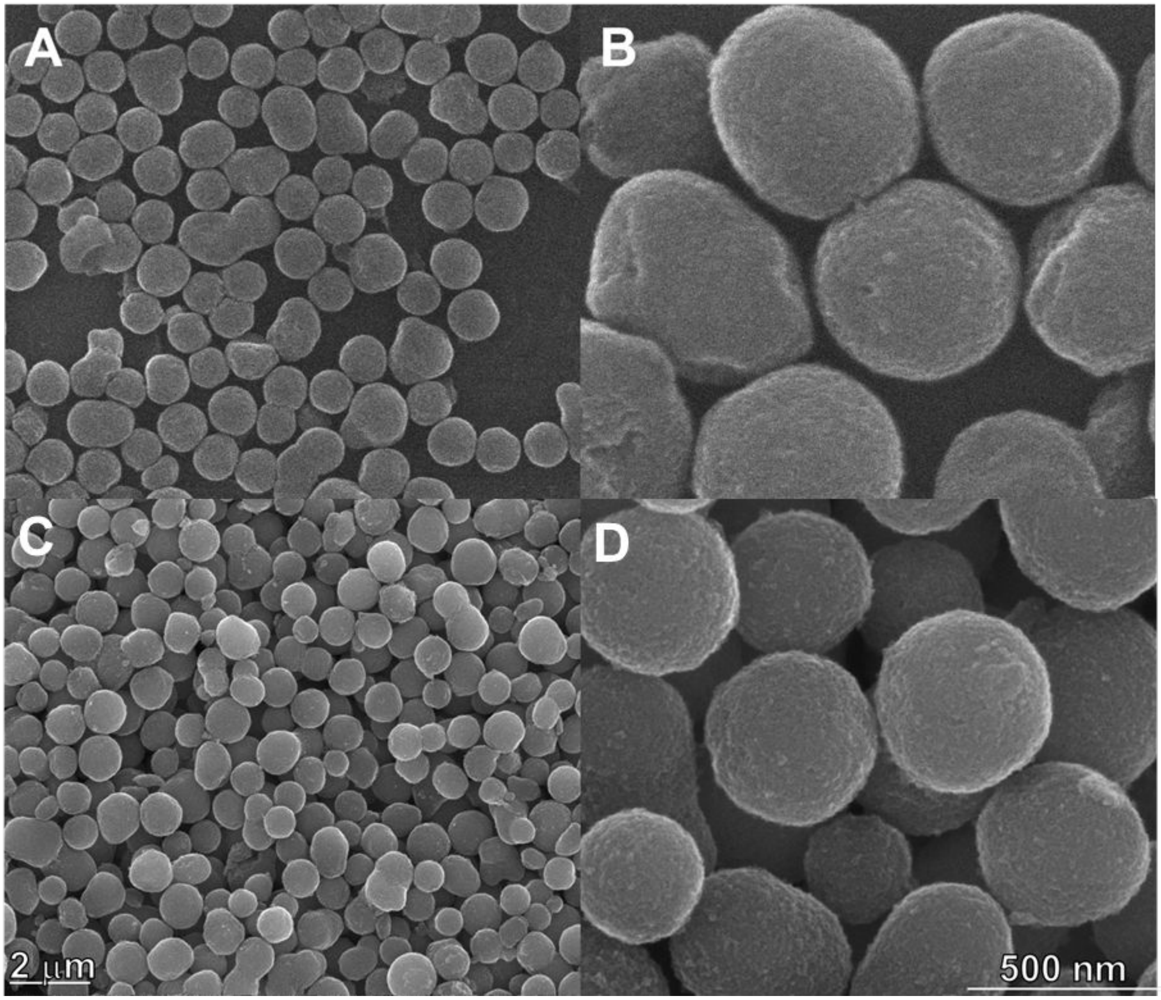

Figure S15. The SEM photographs of PdAu@ACTNS-1 after (A-B) 5 and (C-D) 15 consecutive thermocatalytic FADH runs. Magnification: A, C: 50.000x, B, D: 200.000x

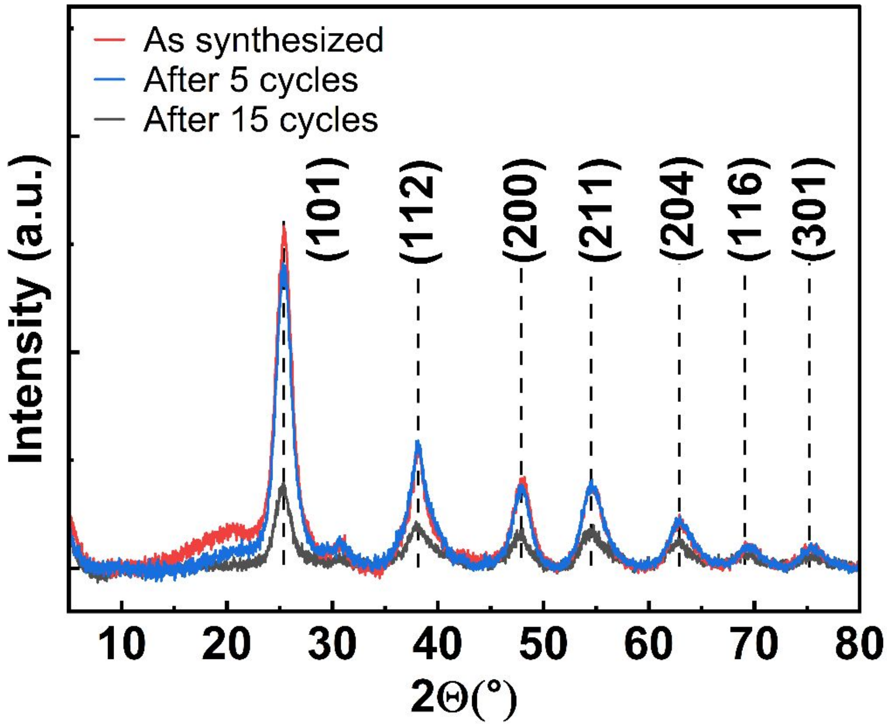

Figure S16. The comparison of XRD spectra of as synthesized and used PdAu@ACTNSs-1 after 5 and 15 consecutive runs.

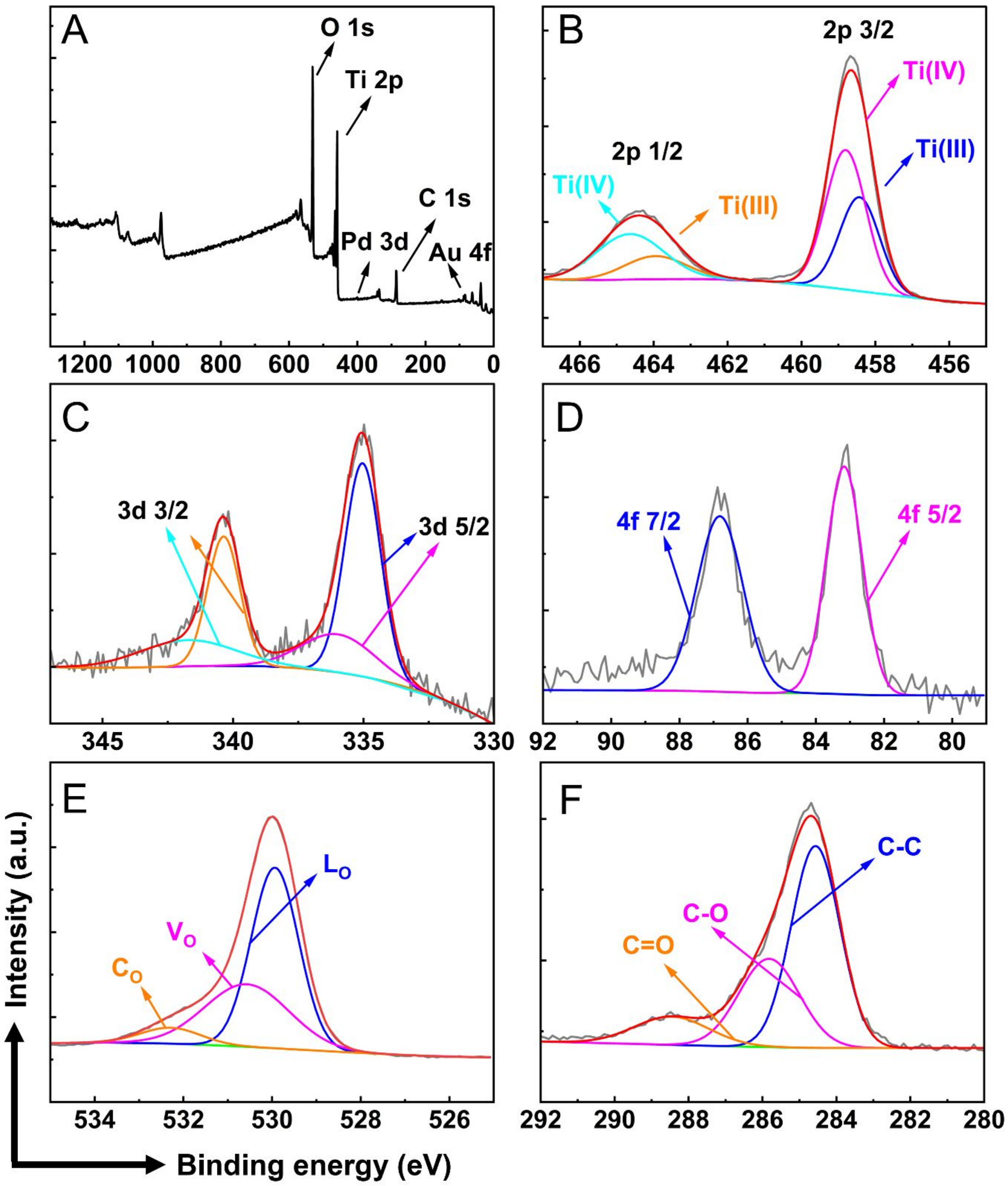

Figure S17. The X-ray photoelectron spectra of used PdAu@ACTNSs-1 after 5 consecutive runs. (A) Survey XPS spectrum, Core level spectra for (B) Ti 2p, (C) Pd 3d, (D) Au 4f, (E) O 1s and (F) C 1s spectra.

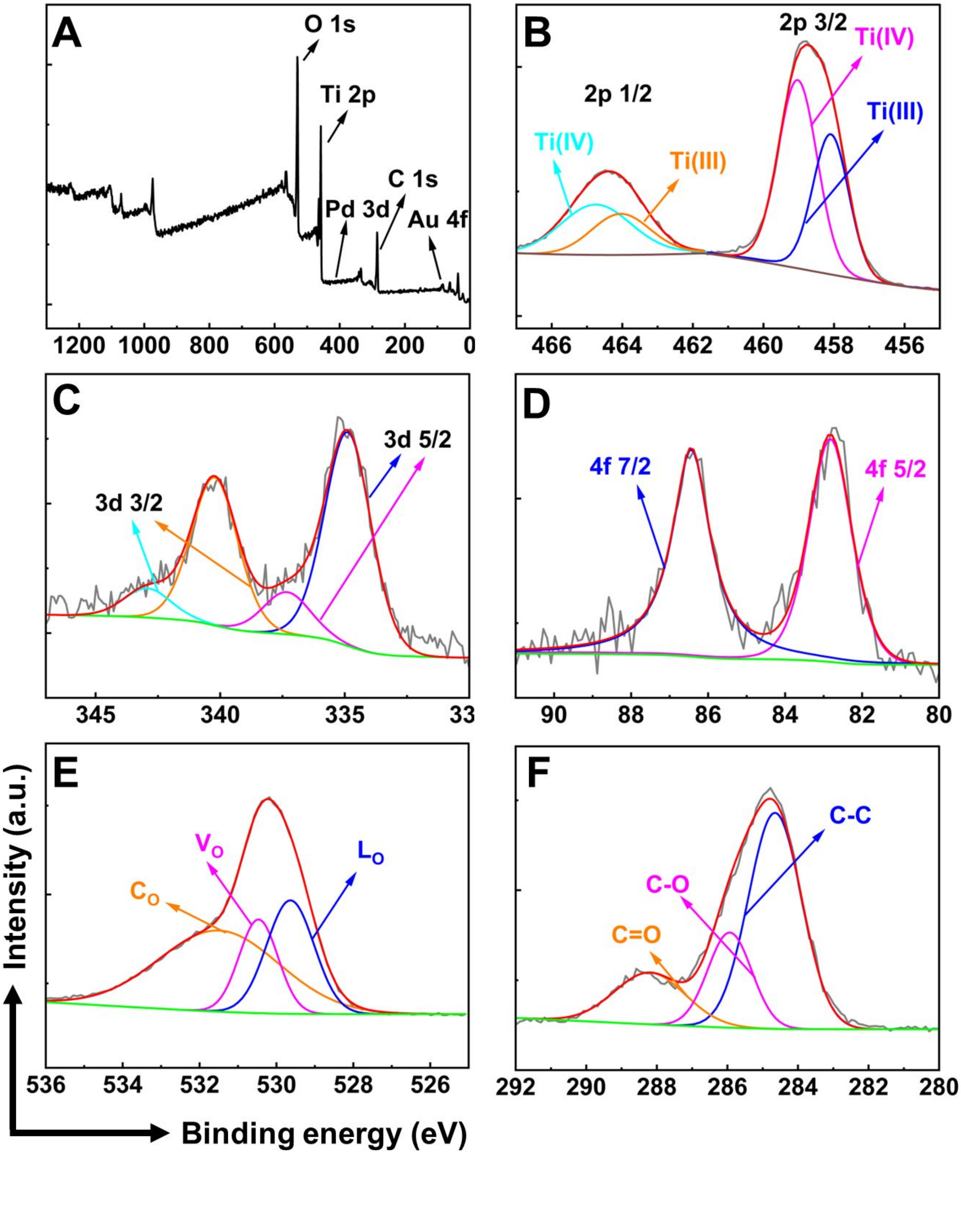

A
O 1s
Ti 2p
C 1s
Pd 3d
Au 4f
1200
1000
800
600
400
200
0
B
2p 3/2
Ti(IV)
2p 1/2
Ti(III)
Ti(IV)
Ti(III)
466
464
462
460
458
456
C
3d 5/2
3d 3/2
345
340
335
33
D
4f 7/2
4f 5/2
90
88
86
84
82
80
E
V_O
L_O
C_O
536
534
532
530
528
526
F
C-C
C-O
C=O
292
290
288
286
284
282
280
Intensity (a.u.)
Binding energy (eV)

Figure S18. The X-ray photoelectron spectra of used PdAu@ACTNSs-1 after 15 consecutive runs. (A) Survey XPS spectrum, Core level spectra for (B) Ti 2p, (C) Pd 3d, (D) Au 4f, (E) O 1s and (F) C 1s spectra.

Table S3. TOF values obtained with different PdAu@ACTNSs-1 concentrations in the visible light driven photocatalytic FADH reaction at 25 ˚C.

Catalyst	Temperature (°C)	TOF[a] (h^{-1})	Reference
PdAu@ACTNSs-1	25	1507.7	This work
PdAu@TiO_2	24	1391.0	S2
Au1Pd2/GO	25	954.2	S3
AuPd–MnOx /ZIF-8–rGO	25	382.1	S4
UiO-66$(COOH)_2$-Cu	25	550	S5
Au_1Pd_2/GO	25	954.2	S6
COP/Pd-Au	25	260.5	S7

a: Initial TOF calculated as 20% of FA conversion.

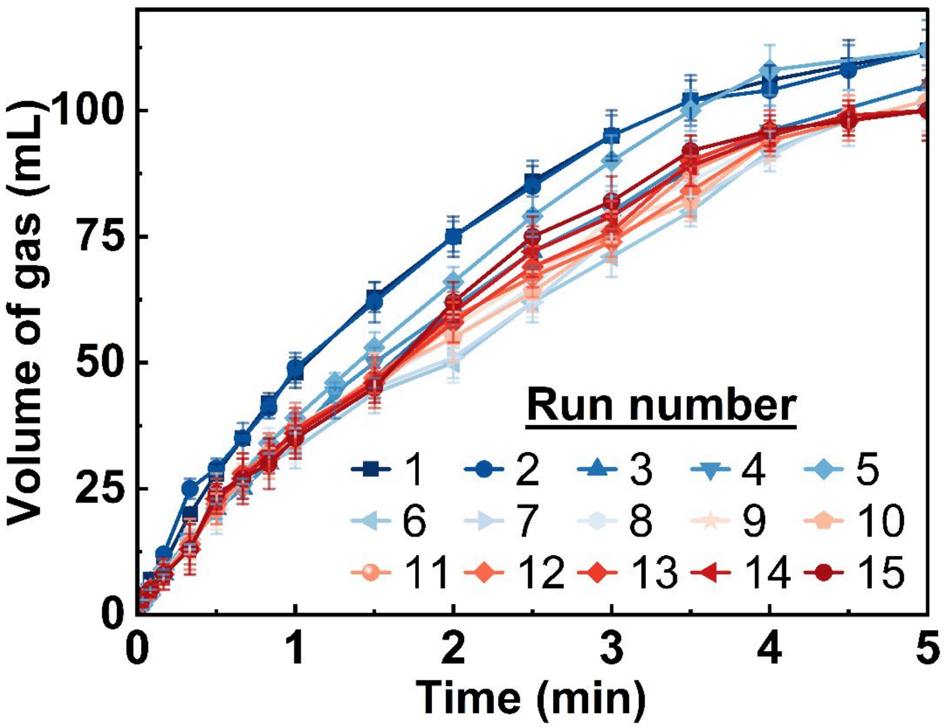

Figure S19. The reusability of PdAu@ACTNSs-1 in visible light driven photocatalytic FADH reaction. Conditions: Pd/Au weight ratio: 1/1, PdAu loading 5.0% wt./wt., FA/SF mole ratio:1/1, PdAu@ACTNSs-1 concentration: 30 mg mL^{-1}, temperature 25 ^{o}C, stirring rate: 350 rpm under visible light.

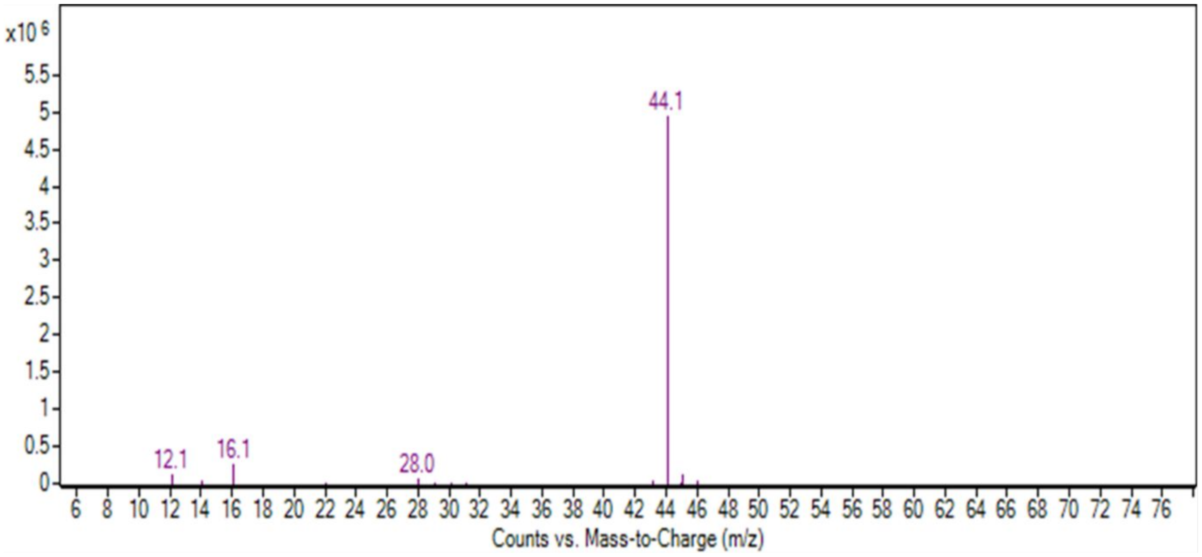

Figure S20. GC-MS analysis spectrum for evolved gas sample in the photocatalytic FADH reaction using PdAu@ACTNSs-1. Conditions: Pd/Au weight ratio: 1/1, PdAu loading 5.0% wt./wt., FA/SF:1/1, PdAu@ACTNSs-1 concentration: 30 mg mL^{-1}, temperature 25 ^{o}C, stirring rate: 350 rpm

References for Supporting Information

(S1) Li, H.; Zhong, J.; Tang, X.; Li, Z.; Ou, W.; Zhou, B.; Wang, C.; Pan, J.; Chen, Y.; Wu, H.; Lu, J.; Li, Y. Y. Band-gap reduction of oxides via partial hydrolysis-enhanced carbothermic reaction. *Appl. Mater. Today* **2023**, *33*, 101864. https://doi.org/10.1016/j.apmt.2023.101864.

(S2) Al-Qurahi, M. A. A.; Demir, M. C.; Tümer, B.; Gökçal, B.; Tuncel, A. Formic Acid Dehydrogenation Catalyzed by Bimetallic Nanoalloys Supported by Monodisperse-Porous Microspheres: Catalytic and Visible Light Driven Photocatalytic Hydrogen Generation. *Int. J. Hydrogen Energy* **2024**, *51*, 111–132. https://doi.org/10.1016/j.ijhydene.2023.09.289.

(S3) Wang, Q.; Lin, S.; Luo, H.; Yu, W.; Liu, W.; Chen, F.; Cheng, D. Enhanced Photocatalytic Dehydrogenation of Formic Acid over Ultrafine Electron-Deficient Pd Nanoparticles Immobilized on Amine-Functionalized Mesoporous Titanium Dioxide. *Int. J. Hydrogen Energy* **2024**, *77*, 1307–1316. https://doi.org/10.1016/j.ijhydene.2024.06.226.

(S4) Yuan, X.; Hu, L.; Zaidi, A. A. S.; Shi, R.; Liu, Y.; Zhang, J.; Zhu, Y.; Wang, J. Oxygen Vacancy-Rich NH2-W18O49 Supported PdAu Catalyst with Enhanced Hydrogen Spillover for Formic Acid Dehydrogenation and CO2 Hydrogenation. *Appl. Catal. B* **2025**, *378*, 125627. https://doi.org/10.1016/j.apcatb.2025.125627.

(S5) Jiang, S.; Yang, J.; Zhai, S.; Zhang, L.; Tu, R.; Yu, T.; Zhai, D.; Sun, L.; Deng, W.; Ren, G. Ambient Hydrogen Storage and Release Using CO $_2$ and an l-Arginine-Functionalized PdAu Catalyst via PH Control. *ACS Catal.* **2022**, *12* (22), 14113–14122. https://doi.org/10.1021/acscatal.2c03893.

(S6) Zhang, Z.; Cao, S.-W.; Liao, Y.; Xue, C. Selective Photocatalytic Decomposition of Formic Acid over AuPd Nanoparticle-Decorated TiO2 Nanofibers toward High-Yield Hydrogen Production. *Appl. Catal. B* **2015**, *162*, 204–209. https://doi.org/10.1016/j.apcatb.2014.06.055.

(S7) Nouruzi, N.; Dinari, M.; Gholipour, B.; Mokhtari, N.; Farajzadeh, M.; Rostamnia, S.; Shokouhimehr, M. Photocatalytic Hydrogen Generation Using Colloidal Covalent Organic Polymers Decorated Bimetallic Au-Pd Nanoalloy (COPs/Pd-Au). *Mol. Catal.* **2022**, *518*, 112058. https://doi.org/10.1016/j.mcat.2021.112058.
